# Performance of Large Language Models on the Brazilian National Medical Education Examination: Comparative Benchmark Study

**DOI:** 10.2196/89839

**Published:** 2026-05-29

**Authors:** Francys de Luca Fernandes da Silva, Eduardo Augusto Roeder, João Victor Bruneti Severino, Matheus Nespolo Berger, Pedro Angelo Basei de Paula, Davi Ferreira, Maria Han Veiga, Thyago Proença de Moraes, Gustavo Lenci Marques

**Affiliations:** 1 R. Imac. Conceição, 1155 - Prado Velho Pontifícia Universidade Católica do Paraná Curitiba, Paraná Brazil; 2 Universidade Federal do Paraná Curitiba, Paraná Brazil; 3 Instituto Tecnológico de Aeronáutica São José dos Campos, São Paulo Brazil; 4 The Ohio State University Columbus, OH United States

**Keywords:** artificial intelligence, benchmarking, Brazilian Portuguese, clinical reasoning, ENAMED, Exame Nacional de Avaliação da Formação Médica, large language models, medical education

## Abstract

**Background:**

Large language models (LLMs) are rapidly incorporated into medical education and examination preparation; yet, most benchmarking evidence is derived from English-language material. Whether frontier commercial models and Brazilian Portuguese domain-specialized systems perform equivalently on high-stakes Brazilian medical examinations remains unclear.

**Objective:**

This study aims to quantify and compare the performance of 9 frontier commercial LLMs and 1 Brazilian Portuguese domain-specialized system (Charcot, Voa Health) on 2026 Brazilian National Medical Education Examination (Exame Nacional de Avaliação da Formação Médica [ENAMED] 2026) and to describe the patterns of systematic between-model error as complementary quality signal.

**Methods:**

All 100 items of ENAMED 2026 (99 valid after annulment) were administered to 10 frontier-panel models across 5 independent runs under identical Portuguese prompts (temperature=0; top-p=.95). Commercial models were accessed through a unified OpenRouter client layer (DeepSeek provider-pinned). The primary outcome was mean accuracy against the preliminary key; the secondary outcomes were convergence error (CE), normalized mean response time (NMRT), and intermodel agreement. Accuracy was analyzed with Shapiro-Wilk, Levene, Kruskal-Wallis (ε^2^), Dunn-Holm post hoc, and a binomial generalized linear mixed model with question and run random intercepts. NMRT excluded Charcot (different stack) and Grok 4 (latency outlier). A total of 7 open-weight and small language models were assessed as a small language model (SLM) substudy.

**Results:**

Frontier-panel accuracy ranged from 73.74% (365/495) for GPT-4o-mini to 96.97% (480/495) for Charcot. Accuracy was nonnormal (Shapiro-Wilk, W=0.82; *P*<.001) with homogeneous variance (Levene *P*=.26). Kruskal-Wallis showed large between-model differences (H_9_=47.65; *P*<.001; ε^2^=0.97). Dunn-Holm flagged 8 of 45 pairs: Charcot was separable from GPT-4o-mini, DeepSeek v3.2-exp, and Grok 4, but not from the top frontier cluster. The generalized linear mixed model preserved the ranking (all comparators odds ratio<1 vs Charcot; upper CI<1 except GPT-5). A total of 9 items met the default CE criterion; item 77 showed 10-of-10 convergence, later confirmed by Instituto Nacional de Estudos e Pesquisas Educacionais Anísio Teixeira rectification, and sensitivity analysis preserved the CE set (5-20 items). Intermodel agreement was high (Fleiss κ=0.852; Krippendorff α=.852). Among 8 retained commercial models, NMRT correlated positively with accuracy (Spearman ρ=0.74; *P*=.04); these results are interpreted as descriptive of an orchestration-level latency–accuracy law. SLM accuracy ranged from 47.47% (Gemma 3 4B) to 82.22% (GPT-OSS 120B), with lower agreement (Fleiss κ=0.508) and 17 CE items. The best SLM-panel model lagged every frontier-panel model except GPT-4o-mini, with a 12-15 percentage-point gap against the top cluster.

**Conclusions:**

On ENAMED 2026, a Brazilian Portuguese domain-specialized system ranked first, indistinguishable from frontier commercial cluster and above subfrontier and open-weight systems. Charcot’s architecture is not publicly disclosed; these findings should be interpreted as comparative black-box evidence of performance and not as mechanistic evidence of specialization. CE was stable, and it prospectively flagged 1 rectified item, supporting its use as a quality assurance screen.

## Introduction

Contemporary clinical judgment requires rational analysis amid a growing body of scientific evidence. Large language models (LLMs) have emerged as candidate tools to support this work, owing to their ability to integrate multiple sources and maintain stable behavior across long interactions, free from the fatigue and working memory constraints that limit human performance. Understanding how reliably LLMs address complex clinical tasks is therefore a pivotal question for their safe adoption in real-world scenarios [1].

Licensing and certification examinations based on multiple-choice questions (MCQs) are widely used as benchmarks. Most published evidence, however, relies on Anglophone exams such as the United States Medical Licensing Examination, limiting extrapolation to health care systems with distinct terminology, epidemiology, and guidelines, including Brazil [2-5].

Recent work evaluating generalist models on Brazilian national examinations (the Revalida and the Brazilian Society of Cardiology board certification exam) has shown inconsistent performance, suggesting that language and local clinical context influence results. In parallel, domain-specific adaptations such as fine-tuning and retrieval-augmented generation over national guidelines have been associated with improvements in clinical reasoning [6-9].

The National Examination for the Evaluation of Medical Education (Exame Nacional de Avaliação da Formação Médica [ENAMED]), established by the Instituto Nacional de Estudos e Pesquisas Educacionais Anísio Teixeira (INEP), comprises MCQs spanning multiple specialties and is aligned with Brazilian national guidelines. ENAMED is therefore a standardized national testbed for both generalist and context-adapted LLMs [10].

In this study, we compared 9 frontier generalist LLMs and 1 Brazilian Portuguese domain-specialized system (Charcot, Voa Health) on ENAMED 2026. We evaluated accuracy, temporal behavior (wall clock), intermodel agreement, and patterns of shared error. Our a priori hypothesis was that regional/domain specialization would be associated with higher accuracy on context-dependent items and that between-model error convergence could identify both shared model blind spots and potential answer-key inconsistencies. We also conducted a secondary subanalysis on 7 open-weight and small language models (SLMs) to characterize the gap between frontier and subfrontier systems in Brazilian Portuguese board-level medical reasoning.

## Methods

### Ethical Considerations

This study did not involve human participants or identifiable data; it used publicly available ENAMED 2026 examination items published by INEP. Institutional review board approval was therefore not applicable.

### Study Design

**Figure 1:**
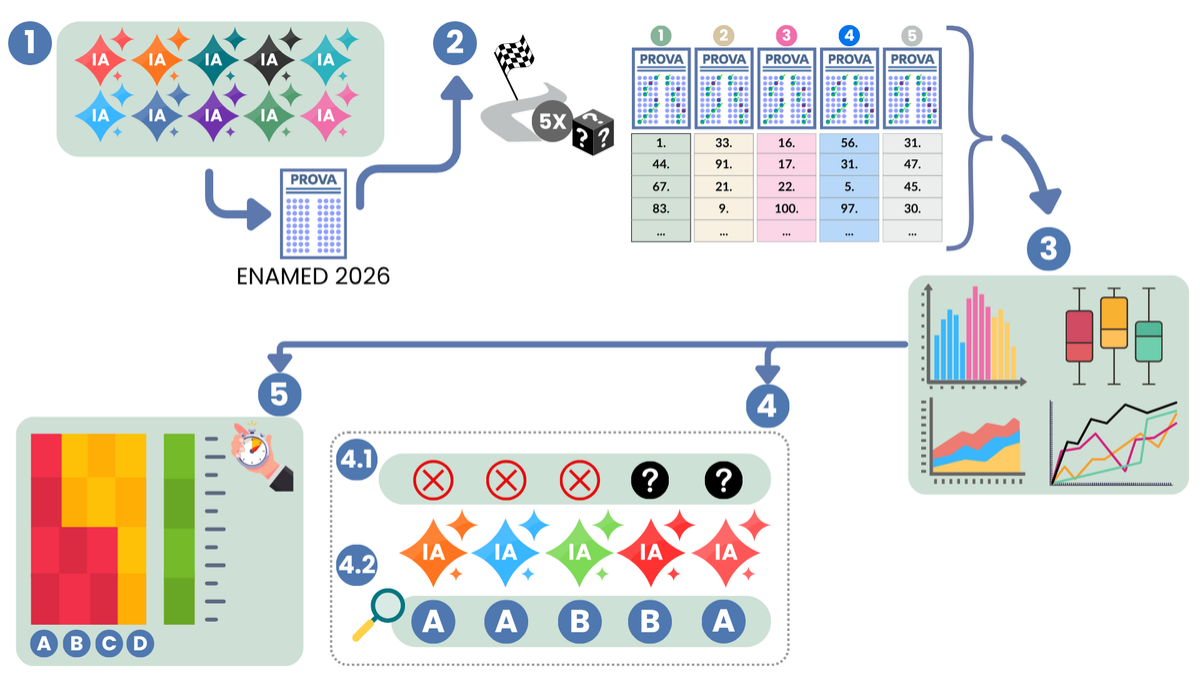
Study workflow for Exame Nacional de Avaliação da Formação Médica (ENAMED) 2026. A total of 10 models (frontier panel) plus 7 small language model (SLM)–panel models were administered all 100 items (99 valid) over 5 runs each, under temperature=0.0 and top-p=0.95, with per-run randomization of item order and alternative order. Analyses: accuracy (primary); convergence error, NMRT, intermodel agreement, and per-question false discovery rate (FDR)–corrected chi-square (secondary); and generalized linear mixed model (GLMM) as sensitivity analysis.

This observational quantitative benchmark administered the ENAMED 2026 examination to 10 LLMs under identical prompting, inference parameters, and orchestration. The study did not involve human participants or identifiable data; it used publicly available ENAMED 2026 items released by INEP. Institutional review board approval was therefore not applicable. Figure 1 summarizes the construction of the study design.

### Evaluated Models

We selected 10 models for the frontier panel (Table 1). Charcot is a proprietary Brazilian Portuguese domain-specialized system. The remaining 9 are frontier commercial application programming interfaces (APIs) accessed through a unified client layer. A total of 7 additional open-weight and SLMs were evaluated under the same protocol as a secondary subanalysis (Table 1 for panel assignment). Model identifiers, version strings, and access windows are reported in Multimedia Appendix 1.

**Table 1:** Evaluated models. Every model other than Charcot was accessed through a unified OpenRouter client layer resolving to the provider’s canonical first-party endpoint (DeepSeek provider-pinned; details provided in the “Methods” section and Multimedia Appendix 1).

Model	Panel^a^
Charcot	Frontier^b^
GPT-5	Frontier
Claude Opus 4.1	Frontier
Claude Sonnet 4.5	Frontier
Gemini 2.5 Pro	Frontier
Grok 4	Frontier
GPT-4.1	Frontier
GPT-4o	Frontier
GPT-4o-mini	Frontier
DeepSeek v3.2-exp	Frontier
GPT-OSS 120B	SLM^c^
Llama 4 Scout	SLM
Qwen3 8B	SLM
Gemma 3 27B	SLM
Ministral 3B	SLM
Phi-4	SLM
Gemma 3 4B	SLM

^a^Indicates analytical panel assignment.

^b^Primary hypothesis panel.

^c^SLM: small language model (subanalysis of open-weight and SLMs).

### Model Access and Reproducibility

All models other than Charcot were accessed through a unified API gateway (OpenRouter [11]) configured to resolve every request to a fixed canonical provider endpoint (OpenAI, Anthropic, Google, xAI, DeepSeek, Meta, Mistral, Microsoft, Alibaba) using a standardized client-side interface. OpenRouter was chosen to equalize client-side orchestration, parameter serialization (temperature, top-p, and seed where exposed), retry/timeout handling, and response parsing across providers, thereby reducing implementation variance between models.

For first-party proprietary models, OpenRouter is methodologically equivalent to a unified client library: the model weights, inference infrastructure, and version exposed are those of the provider itself and do not cache chat completions by default. Additionally, each of the 5 runs used independent seeds and randomized question and alternative order, making response-level cache hits improbable regardless of the caching configuration. Specific OpenRouter model identifiers, version pins, access windows, and the underlying provider routing for each model are listed in Multimedia Appendix 1.

Among the evaluated systems, DeepSeek v3.2-exp is the only model for which OpenRouter exposes multiple inference providers. To prevent uncontrolled provider rotation from introducing silent infrastructure variance between runs, the DeepSeek route was pinned to a single provider for the entire study window, with the resolved provider identifier, routing decision, and per-call headers logged for every request. Provider logs and configuration are retained in Multimedia Appendix 2. For all other commercial models in the frontier panel, OpenRouter resolves to the provider’s canonical first-party end point, so provider pinning is not applicable.

Charcot is a proprietary system developed by Voa Health. For competitive and regulatory reasons, its architecture, training corpus composition, retrieval components, and inference pipeline cannot be disclosed at the level of granularity available for other models. From a methodological standpoint, Charcot can be interpreted as a black-box system under evaluation, subjected to the same operational conditions as the commercial APIs (single final answer plus rationale, identical Portuguese prompts, no external browsing, and deterministic inference parameters where exposed). This opacity is an inherent limitation of including proprietary systems in benchmarks and is properly discussed in the “Study Limitations” section.

All inference calls were executed on a single orchestrator host (specifications are available in Multimedia Appendix 1). External browsing and tool use were disabled for every model. Scoring was automated against the official preliminary key to eliminate assessor subjectivity.

### ENAMED 2026 Examination

The official ENAMED 2026 examination (Caderno 01, exam type 1) was used. It comprises 100 MCQs, each with a single correct answer among 5 alternatives (A-E), spanning internal medicine, surgery, primary care, public health, pediatrics, and obstetrics and gynecology. Items and alternatives were transcribed verbatim into structured text. The preliminary official answer key released by INEP was adopted as the gold standard. Item 10 was annulled by INEP after release, yielding 99 valid items. Item 77 was subsequently rectified by INEP (key D to B); primary accuracy was computed against the preliminary key used at analysis time, and the postrectification interpretation of item 77 is provided as a secondary contextual analysis in the “Convergence Error” section.

### Examination Administration Protocol

Each model completed the full examination in 5 independent runs, producing 500 responses per model (100 items × 5 runs). In every run we applied (1) question-order randomization preserving content integrity, (2) internal randomization of alternatives (A-E) preserving the text bound to each option, (3) fixed pseudorandom seeds per run for reproducibility, and (4) standardized system and user prompts in Portuguese requesting a single final alternative plus a concise justification. For every item, we recorded the selected alternative, the generated justification, and the end-to-end wall-clock response time (from request submission to full response receipt).

Inference parameters were held constant across models wherever exposed by the API: temperature=0.0, top-p=0.95. Temperature=0.0 reduces output variability but does not guarantee full determinism, because of hardware nondeterminism (CUDA kernel reductions, atomic adds, and mixed precision) and tied token probabilities [12-14]. The 5-run protocol was therefore chosen to empirically capture residual stochastic variability rather than to force strict determinism; between-run SDs per model are reported in Results (Frontiers in Multimedia Appendix 3 and SLM in Multimedia Appendix 4). Prompt templates (including exploratory reasoning-oriented variants tested during piloting and not used in the primary analysis) are provided in Multimedia Appendix 1.

### Outcomes

#### Primary Outcome: Accuracy

The primary outcome was mean accuracy per model, defined as the proportion of valid items answered correctly relative to the preliminary official answer key, averaged over the 5 runs.

#### Secondary Outcome: Convergence Error

Convergence error (CE) is a construct introduced in this work to capture systematic shared error between models. A question was flagged as CE-positive when at least 3 evaluated models converged on the same incorrect alternative in all 5 runs (default threshold: ≥3 models × 5 of 5 runs). A sensitivity grid spanning {2, 3, 4} model thresholds × {3, 4, 5} run thresholds was prespecified to assess CE set stability. CE is a descriptive signal; no inferential test was attached to it.

#### Secondary Outcome: Normalized Mean Response Time

Wall-clock time per item was averaged across the 5 runs for each model and question, then standardized as a *z* score against (1) the global mean and SD (Normalized Mean Response Time [NMRT]-global) and (2) the within-model mean and SD (NMRT-model). NMRT reflects end-to-end orchestration latency (routing, provider queueing, inference, and streaming), rather than pure inference time. Two prespecified exclusions were applied to NMRT. Charcot was excluded because it does not traverse the OpenRouter stack used for every other commercial model, so absolute wall-clock comparisons would be structurally nonequivalent. Grok 4 was excluded because its xAI-via-OpenRouter latency distribution showed an extremely long tail (mean 35.6, SD 33.6 s; median 22.6, IQR 18.2-37.7 s; occasional spikes exceeding 400 s) that dominated the variance of the global *z* score and distorted visual comparisons among the remaining, more homogeneously-routed models.

#### Secondary Outcome: Intermodel Agreement

Agreement on selected alternatives across the evaluated models was quantified with Fleiss κ and Krippendorff α (nominal) over 99 valid items, providing a complementary view of between-model convergence that is symmetric with respect to correctness (both shared right and shared wrong answers increase agreement).

#### Qualitative Analysis of Rationales

For CE-positive items and items of particular clinical interest, we conducted a descriptive qualitative analysis of the generated justifications aimed at (1) identifying correct reasoning leading to incorrect conclusions (or vice versa), (2) mapping alignment with Brazilian national guidelines, and (3) characterizing recurrent reasoning shortcuts or omissions. No formal faithfulness metric was applied; this analysis contextualizes the numerical findings rather than quantifying them.

### Statistical Analysis

All analyses were conducted in Python 3.13 using scipy, statsmodels, scikit-posthocs, and purpose-built modules for CE, NMRT, and agreement. Accuracy distributions were checked for normality with the Shapiro-Wilk test and for variance homogeneity with the Levene test. Because normality was rejected, between-model differences were tested with the Kruskal-Wallis rank-sum test; effect sizes were quantified as epsilon squared and eta-squared-H, interpreted following Tomczak and Tomczak [15]: small, 0.01-0.06; moderate, 0.06-0.14; and large, ≥0.14. When the omnibus test was significant, pairwise comparisons followed with Dunn test under Holm correction, using rank-biserial correlation magnitude as the pair-level effect size with direction given by mean ordering. Per-question heterogeneity was assessed with chi-square tests pooled across runs under Benjamini-Hochberg false discovery rate (FDR) control at α=.05. As a prespecified sensitivity analysis, a binomial GLMM with logit link modeled per-item correctness as a function of model (fixed effect, Charcot as reference) with crossed random intercepts for question and run, producing item- and run-conditioned odds ratios (ORs) with 95% CIs. Between-run variability was summarized as SD [16], coefficient of variation, and range in percentage points (pp) per model. The Spearman correlation between mean NMRT and mean accuracy was computed over the subset of models retained for NMRT analysis. The significance threshold for all inferential tests was α=.05; CE and agreement analyses were descriptive.

### Open-Weight and SLMs Substudy

A total of 7 additional systems were evaluated under the same protocol as an SLM substudy: 4 open-weight mid/large models (GPT-OSS 120B, Llama 4 Scout, Qwen3 8B, and Gemma 3 27B) and 3 SLMs (Ministral 3B, Phi-4, and Gemma 3 4B). Selection prioritized models that (1) were publicly released with open weights, (2) are representative of current open-weight frontiers at distinct capacity tiers (3B to 120B parameters), and (3) were accessible through the same OpenRouter client layer used for the frontier panel, ensuring identical prompting, decoding (temperature=0.0; top-p=0.95), 5-run structure, question and alternative randomization, and scoring. OpenRouter IDs, underlying providers, version pins, and access windows for each SLM-panel model are listed in Multimedia Appendix 1. This panel was analyzed independently from the frontier hypothesis test, with its own Kruskal-Wallis, CE, agreement, and run-variability outputs, to avoid diluting the primary contrast between frontier commercial models and the Brazilian Portuguese domain-specialized system while still allowing the 2 panels to be read side by side when characterizing the frontier × subfrontier gap.

## Results

### General Model Accuracy

**Figure 2:**
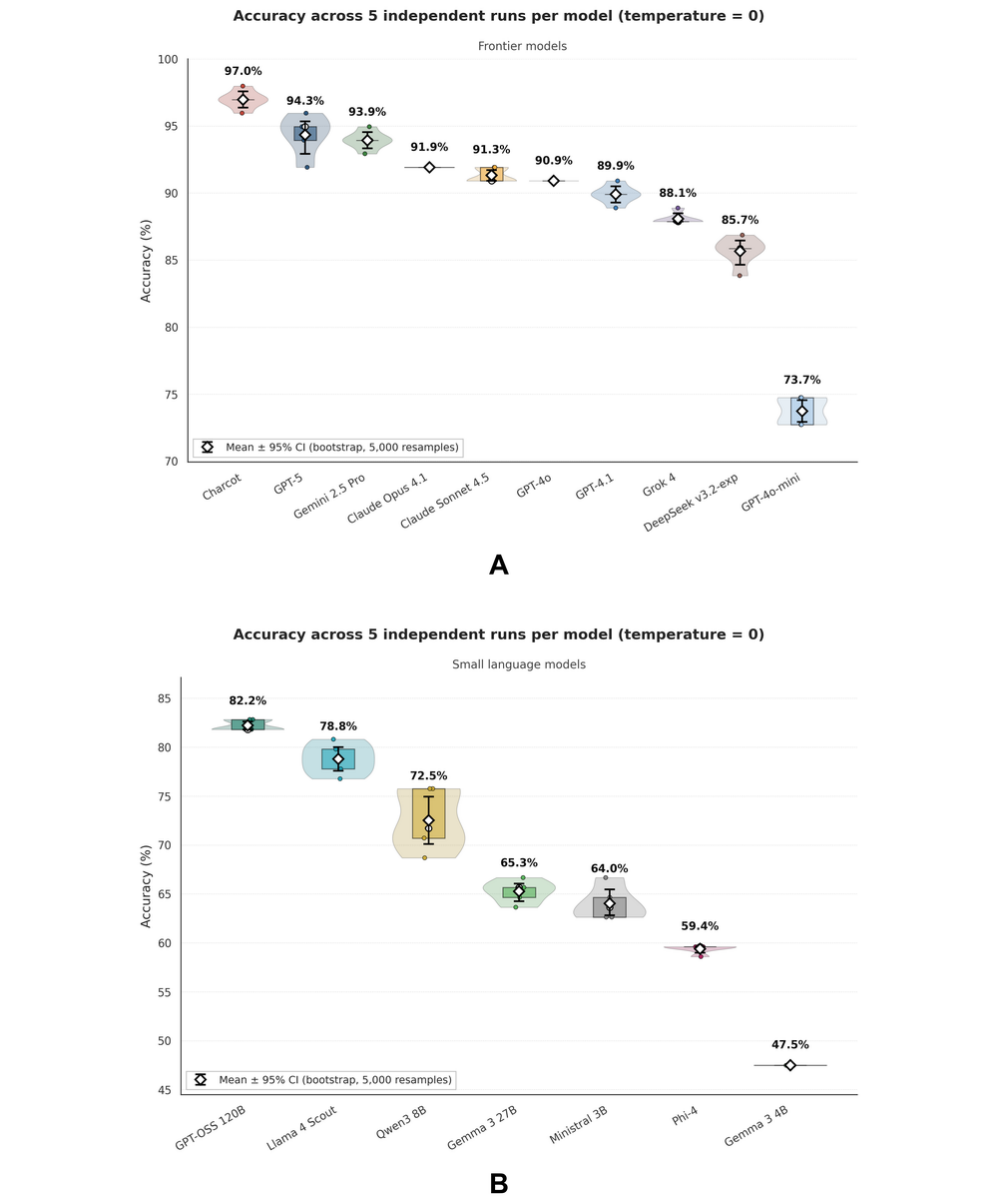
Mean accuracy per model with 95% bootstrap CIs (5000 draws) on Exame Nacional de Avaliação da Formação Médica (ENAMED) 2026 (99 valid items × 5 runs). (A) Frontier panel: 10 models (Charcot and 9 frontier commercial application programming interfaces [APIs]). (B) Small language model (SLM) substudy: 7 open-weight and SLMs. Error bars reflect between-run variability. Between-run SD stayed ≤1.53 percentage points (pp) in every frontier-panel model (median 0.71, IQR 0.48-0.94 pp) and ≤3.15 pp in every SLM-panel model, supporting the 5-run protocol. Detailed run-variability plots and quantile-quantile diagnostics supporting the use of Kruskal-Wallis in both panels are reported in Multimedia Appendices 3 and 4.

The preliminary official key comprised 99 valid items (item 10 annulled). Across 5 independent runs with temperature=0.0, Charcot achieved the highest mean accuracy (480/495, 96.97%; SD 0.71 pp; 95% CI 96.36%-97.58%), followed by GPT-5 (467/495, 94.34%; SD 1.53), Gemini 2.5 Pro (465/495, 93.94%; SD 0.71), Claude Opus 4.1 (455/495, 91.92%; SD 0.00), Claude Sonnet 4.5 (452/495, 91.31%; SD 0.55), GPT-4o (450/495, 90.91%; SD 0.00), GPT-4.1 (445/495, 89.90%; SD 0.71), Grok 4 (436/495, 88.08%; SD 0.45), DeepSeek v3.2-exp (424/495, 85.66%; SD 1.11), and GPT-4o-mini (365/495, 73.74%; SD 1.01) (Table 2 and Figure 2A). Within-model SD stayed below 1.53 pp in every model (median 0.71, IQR 0.48-0.94 pp), supporting the adequacy of the 5-run protocol for capturing residual stochastic variability under deterministic decoding (detailed per-model run variability and quantile-quantile diagnostic are available in Multimedia Appendix 3). Accuracy was not normally distributed (Shapiro-Wilk W=0.82; *P*<.001; n=50) but variances were homogeneous (Levene W=1.29; *P*=.26, k=10). Kruskal-Wallis therefore served as the primary between-model test: H_9_=47.65; *P*<.001; ε^2^=0.97; η^2^_H=0.97. The unusually high ε^2^ is a structural property of the design rather than an overstatement: with only 5 replicates per model under deterministic decoding, within-model variance is near 0, so the between-model component accounts for virtually all variance in the pooled rank distribution. We therefore interpret ε^2^ here as a between-model separability index under controlled decoding, and report distribution-level (Dunn-Holm) and item-conditional (GLMM) evidence as complementary, less ceiling-sensitive views.

**Table 2:** Accuracy on Exame Nacional de Avaliação da Formação Médica (ENAMED) 2026, frontier panel (10 models × 5 runs × 99 valid items). We computed 95% CIs by 5000-draw bootstrap. Kruskal-Wallis H9=47.65; *P*<.001; ε2=0.97 (large, details in the “Methods” section).

Model	Accuracy, mean (SD)^a^	Median (IQR); % (pp)	Minimum-maximum (%)	95% CI	Correct/total, n/N
Charcot	96.97 (0.71)	96.97 (96.97-96.97); 0.00	95.96-98.99	96.36-97.58	480/495
GPT-5	94.34 (1.53)	94.95 (93.94-94.95); 1.01	91.92-95.96	92.93-95.35	467/495
Gemini 2.5 Pro	93.94 (0.71)	93.94 (93.94-93.94); 0	92.93-94.95	93.33-94.55	465/495
Claude Opus 4.1	91.92 (0.00)	91.92 (91.92-91.92); 0	91.92-91.92	91.92-91.92	455/495
Claude Sonnet 4.5	91.31 (0.55)	90.91 (90.91-91.92); 1.01	90.91-91.92	90.91-91.72	452/495
GPT-4o	90.91 (0.00)	90.91 (90.91-90.91); 0	90.91-90.91	90.91-90.91	450/495
GPT-4.1	89.90 (0.71)	89.90 (89.90-89.90); 0	88.89-90.91	89.29-90.51	445/495
Grok 4	88.08 (0.45)	87.88 (87.88-87.88); 0	87.88-88.89	87.88-88.48	436/495
DeepSeek v3.2-exp	85.66 (1.11)	85.86 (85.86-85.86); 0	83.84-86.87	84.65-86.46	424/495
GPT-4o-mini	73.74 (1.01)	73.74 (72.73-74.75); 2.02	72.73-74.75	72.93-74.55	365/495

^a^Mean accuracy is expressed in percentage (%), while SD is expressed in percentage points (pp).

### Pairwise Post Hoc Comparisons (Dunn-Holm)

**Figure 3:**
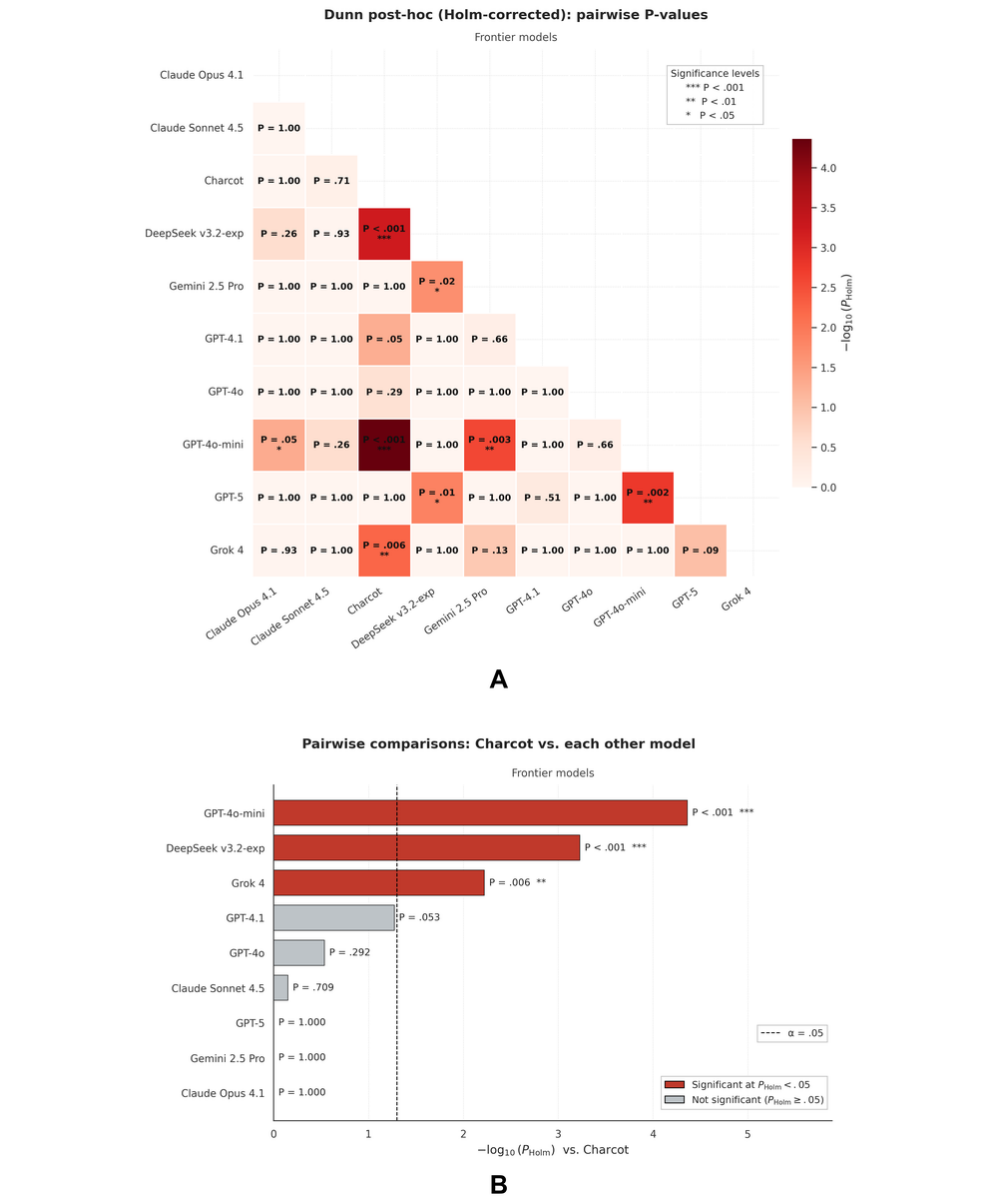
Pairwise post hoc structure (Dunn-Holm). (A) Full Dunn-Holm heatmap of pairwise *P* values across the 10 frontier-panel models; cells below the Holm-corrected .05 threshold are highlighted. Charcot is separable from GPT-4o-mini, DeepSeek v3.2-exp, and Grok 4, but overlaps with the top frontier commercial cluster (GPT-5, Gemini 2.5 Pro, Claude Opus 4.1, Claude Sonnet 4.5, GPT-4o, and GPT-4.1). (B) Charcot-focused detail of the same matrix, restricted to pairs involving Charcot and ordered by adjusted *P* value, to facilitate interpretation of the primary contrast.

Dunn test with Holm correction identified 8 of 45 pairs as significant (*P*<.05; Table 3 and Figure 3). Charcot differed from GPT-4o-mini (*P*=<.001; |*r*|=1.00), DeepSeek v3.2-exp (*P*=<.001; |*r*|=1.00), and Grok 4 (*P*=.006; |*r*|=1.00). GPT-4o-mini was significantly lower than GPT-5 (*P*=.002), Gemini 2.5 Pro (*P*=.003), and Claude Opus 4.1 (*P*=.049). DeepSeek v3.2-exp differed from GPT-5 (*P*=.01) and Gemini 2.5 Pro (*P*=.02). All other pairwise comparisons (including Charcot vs GPT-5, Gemini 2.5 Pro, Claude Opus 4.1, Claude Sonnet 4.5, GPT-4o, and GPT-4.1) did not reach significance after Holm adjustment (*P*≥.05). Rank-biserial magnitudes are reported in Table 3; the direction of each pairwise comparison is given by the mean ordering in Table 2.

**Table 3:** Dunn pairwise post hoc (Holm correction); only pairs with *P*<.05 are shown. Effect sizes are reported as rank-biserial magnitudes (|r|); the direction of each comparison is given by the ordering of mean accuracy in The remaining 37 pairs were nonsignificant after Holm adjustment (P≥.05).

Model A	Model B	*P* (Holm)	Rank-biserial, |*r*|
Charcot	GPT-4o-mini	<.001	1.00
Charcot	DeepSeek v3.2-exp	<.001	1.00
GPT-5	GPT-4o-mini	.002	1.00
Gemini 2.5 Pro	GPT-4o-mini	.003	1.00
Charcot	Grok 4	.006	1.00
GPT-5	DeepSeek v3.2-exp	.01	1.00
Gemini 2.5 Pro	DeepSeek v3.2-exp	.02	1.00
Claude Opus 4.1	GPT-4o-mini	.049	1.00

### Item-Conditional Sensitivity Analysis (GLMM)

**Figure 4:**
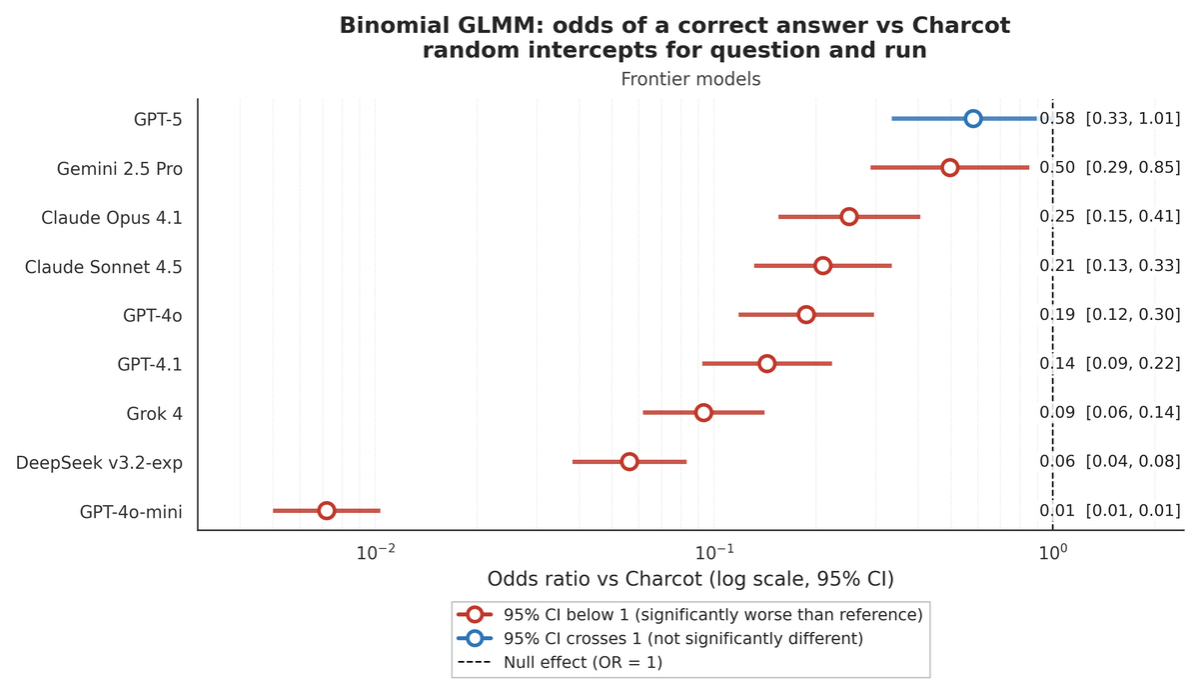
Generalized linear mixed model (GLMM) forest plot of frontier-panel odds ratios (ORs) vs Charcot (reference) with 95% CIs. Question and run were entered as crossed random intercepts. GPT-5 is the only comparator whose CI crosses unity.

The binomial GLMM (details in “Methods” section) absorbed item-level and run-level heterogeneity. Every comparator model had an OR below 1 against Charcot. GPT-5 crossed unity by the upper CI (OR 0.58, 95% CI 0.33-1.01), which was not significant; all other models had upper 95% CIs below 1: Gemini 2.5 Pro OR 0.50 (95% CI 0.29-0.85), Claude Opus 4.1 OR 0.25 (95% CI 0.15-0.41), Claude Sonnet 4.5 OR 0.21 (95% CI 0.13-0.33), GPT-4o OR 0.19 (95% CI 0.12-0.30), GPT-4.1 OR 0.14 (95% CI 0.09-0.22), Grok 4 OR 0.09 (95% CI 0.06-0.14), DeepSeek v3.2-exp OR 0.06 (95% CI 0.04-0.08), GPT-4o-mini OR 0.007 (95% CI 0.005-0.010). The GLMM (Figure 4) therefore preserved the Kruskal-Wallis ranking after conditioning on item difficulty and run effects (Figures 3A and B).

### Per-Question Between-Model Differences

Chi-square tests (model × correct, pooled over runs) followed by Benjamini-Hochberg FDR control at α=.05 identified 34.3% (34/99) of valid items as showing significant between-model differences, considerably above the 5% expected under the global null. The maximum −log₁₀(P) reached 6.97, corresponding to items in which model responses split in a near-binary fashion. This concentration of FDR-significant items supports the substantive rather than spurious nature of between-model differences. The full per-question −log₁₀(P) plot is provided in Multimedia Appendix 3.

### Intermodel Agreement

Agreement on selected alternatives across the 10 frontier-panel models was high: Fleiss κ=0.852 and Krippendorff α=.852 over 99 valid items × 5 runs. The SLM panel (7 models) showed substantially lower agreement (κ=0.508; α=.509), quantifying the greater idiosyncrasy of open-weight and SLMs.

### CE Criterion

**Figure 5:**
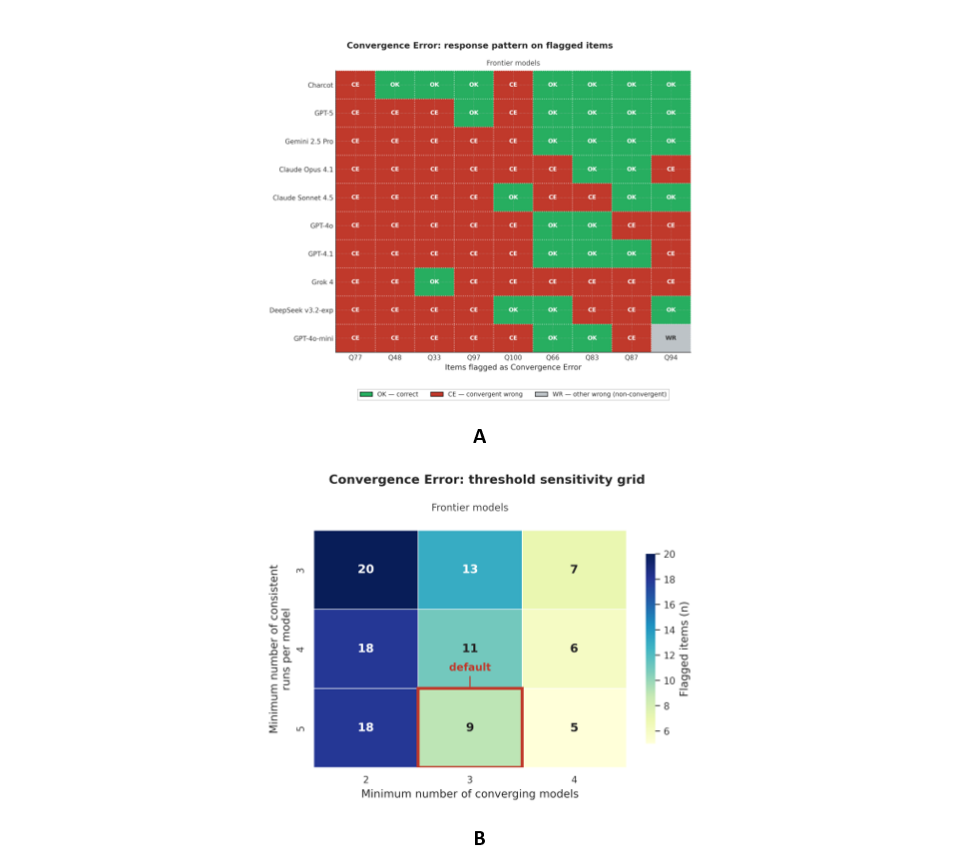
Convergence error (CE; frontier panel). (A) Heatmap across the 10 frontier-panel models for the 9 items flagged at the default threshold (≥3 models × 5 of 5 runs); each cell shows the alternative selected by a model across the 5 runs. Item 77 (all 10 models =B vs preliminary key =D) was subsequently rectified by the Instituto Nacional de Estudos e Pesquisas Educacionais Anísio Teixeira (INEP). (B) Threshold sensitivity grid: number of CE-positive items as a function of model threshold (x-axis) and run threshold (y-axis). The preregistered default is highlighted; the CE set is stable across adjacent configurations.

Using the preregistered default threshold (≥3 models converging on the same incorrect alternative in all 5 runs), 9 of 99 items met the CE criterion in the frontier panel (Q33, Q48, Q66, Q77, Q83, Q87, Q94, Q97, Q100; Table 4 and Figures 5A and B). Item 77 was the strongest case: all 10 frontier-panel models selected alternative B, whereas the preliminary official key was D, consistent with the subsequent INEP rectification to B. Items 48, 33, 97, and 100 showed convergence involving 7 to 8 of 10 models on an alternative other than the preliminary key, and items 66, 83, 87, and 94 showed tighter convergence involving 3 of 10 models.

**Table 4:** Convergence error items at the default threshold (≥3 models × 5 of 5 runs) in the frontier panel.

Item	Preliminary key	Convergent wrong	Models, n/N
77^a^	D^a^	B^a^	10/10
48	D	C	8/10
33	C	A	7/10
97	C	D	7/10
100	A	C	7/10
66	A	B	3/10
83	B	C	3/10
87	B	D	3/10
94	A	C	3/10

^a^Subsequently rectified by the Instituto Nacional de Estudos e Pesquisas Educacionais Anísio Teixeira (INEP; key D to B).

Sensitivity analysis across {2, 3, 4} model thresholds × {3, 4, 5} run thresholds confirmed that the CE set is stable (Table 5 and Figure 3B). The strictest configuration (≥4 models × 5 runs) retained 5 items, all contained within the default set; the most permissive configuration (≥2 models × 3 runs) expanded to 20 items; and the default 9-item set persisted across adjacent configurations.

**Table 5:** Convergence error sensitivity grid: number of flagged items by threshold combination.

Run threshold	Model threshold		
	≥2 models	≥3 models	≥4 models
≥3 of 5 runs	20^a^	13	7
≥4 of 5 runs	18	11	6
≥5 of 5 runs (default)^b^	18	9^c^	5^d^

^a^Corresponds to the most permissive configuration (≥2 models × 3 of 5 runs).

^b^All 5 items flagged under the strictest configuration are contained in the default set.

^c^Corresponds to the preregistered default (≥3 models × 5 of 5 runs).

^d^Corresponds to the strictest configuration (≥4 models × 5 of 5 runs).

### NMRT Analyses

**Figure 6:**
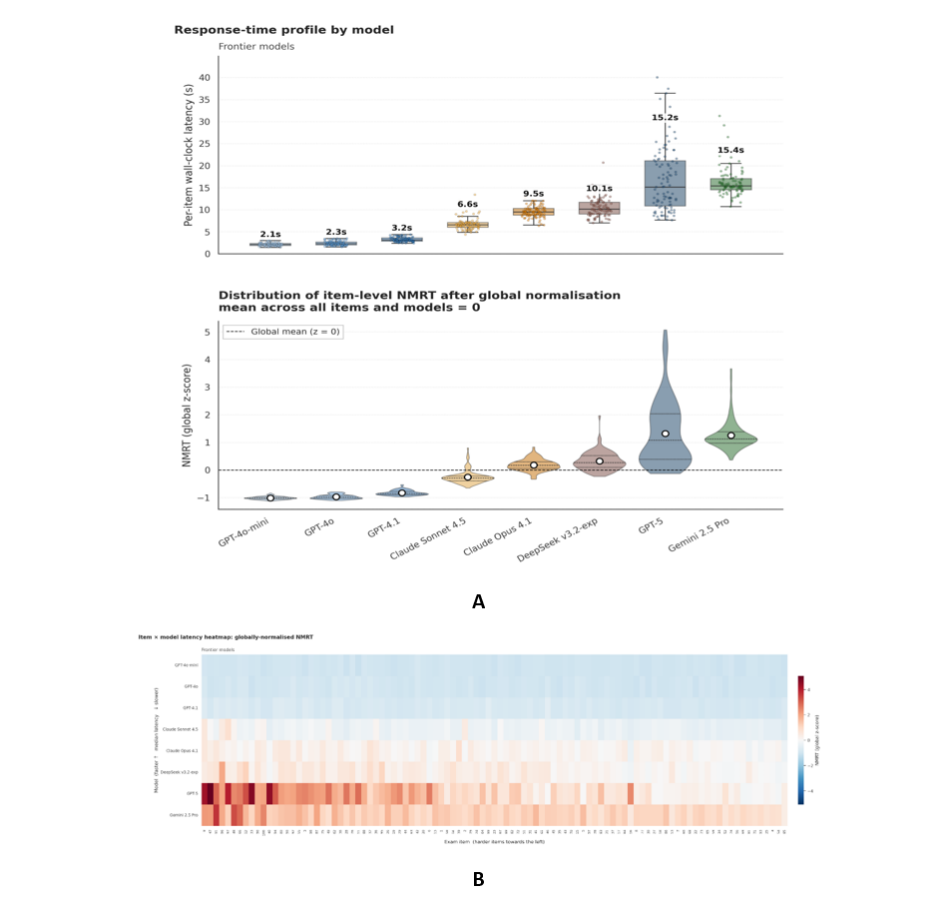
Normalized mean response time (NMRT) frontier panel. (A) NMRT distribution per retained model (violin plot). Charcot and Grok 4 are excluded per prespecification. Reasoning-native models (GPT-5 and Gemini 2.5 Pro) dominate the upper latency tail; nonthinking models cluster near 2-3 s. (B) Per-item NMRT delta heatmap, showing per-model deviation from the item-level NMRT mean, which complements panel A by exposing item-specific latency structure beyond the model-level summary.

NMRT analyses used the 2 prespecified exclusions (Charcot: different stack; Grok 4: long-tail routing latency; details in the “Methods” section). Among the 8 OpenRouter-routed commercial models retained, mean per-question wall-clock time ranged from 2.11 s (GPT-4o-mini) to 16.64 s (GPT-5), with reasoning-native models (GPT-5 and Gemini 2.5 Pro) clustered near 16 s and nonthinking models (GPT-4o-mini, GPT-4o, and GPT-4.1) clustered near 2-3 s (Table 6 and Figure 6). Among the 8 comparably routed models, the reasoning-native systems (GPT-5 and Gemini 2.5 Pro) occupied the upper tails of both the latency and accuracy distributions. A positive Spearman rank correlation was observed between mean NMRT and mean accuracy (ρ=0.74; *P*=.04; n=8); we interpret this observation as descriptive of orchestration behavior (routing, provider queueing, and reasoning-token generation) under the included models rather than as evidence of a general latency–accuracy relationship, given the small n and the prespecified exclusions required to define the analytic set. The correlation also does not survive reinclusion of Grok 4, whose atypical xAI-via-OpenRouter tail compresses the rank statistic. Per-model internal NMRT *z* scores are reported to avoid confounding item difficulty with baseline model speed.

**Table 6:** Secondary outcome, normalized mean response time (NMRT; frontier panel; Charcot and Grok 4 excluded per prespecification). Spearman rank correlation (NMRT × accuracy) ρ=0.74; *P*=.04; n=8.

Model	Wall-clock (s), mean (SD)	Median (IQR) (s)	NMRT global *z* score	Questions timed, n
GPT-4o-mini	2.11 (SD 0.34)	2.09 (1.87-2.33)	−1.01	99
GPT-4o	2.37 (SD 0.49)	2.28 (2.00-2.68)	−0.97	99
GPT-4.1	3.26 (SD 0.55)	3.15 (2.87-3.59)	−0.83	99
Claude Sonnet 4.5	6.81 (SD 1.47)	6.59 (6.01-7.07)	−0.26	99
Claude Opus 4.1	9.56 (SD 1.31)	9.51 (8.78-10.31)	+0.18	99
DeepSeek v3.2-exp	10.41 (SD 2.07)	10.12 (9.04-11.71)	+0.32	99
Gemini 2.5 Pro	16.22 (SD 3.22)	15.41 (14.51-17.07)	+1.25	99
GPT-5	16.64 (SD 7.25)	15.15 (10.87-21.12)	+1.32	99

### Qualitative Analysis of Rationales

For the items with the highest CE or clinical salience signal (Q33, Q48, Q77, Q97, and Q100) we reviewed the generated justifications. In Q33 (postabandonment tuberculosis treatment), Charcot answered correctly in all runs, aligning with Ministry of Health recommendations regarding sensitivity testing and sputum culture in the presence of discordance between the rapid molecular test for tuberculosis and smear microscopy; several generalist models, notably Gemini 2.5 Pro, tended to prematurely restart the basic regimen or repeat the rapid molecular test for tuberculosis as primary management. In Q48 (pleural effusion of probable tuberculous etiology), Charcot prioritized bedside ultrasound and diagnostic thoracentesis in most runs, whereas GPT-5 and Gemini 2.5 Pro concentrated on computed tomography and chest drainage strategies that did not match the expected answer. In Q77 (cultural health knowledge in indigenous populations), all frontier-panel models selected alternative B, later confirmed by INEP rectification. For Q97 (familial short stature), Charcot answered correctly in all runs but occasionally conflated elements of familial short stature and constitutional delay of growth and puberty in its justifications, while Gemini 2.5 Pro and GPT-5 consistently classified the condition as constitutional delay. Finally, in Q100 (criteria for high-risk prenatal care), DeepSeek v3.2-exp and Claude Sonnet 4.5 correctly prioritized third-trimester malaria, while most of the other models (including Charcot in 4 of 5 runs) converged on dichorionic diamniotic twin pregnancy [17].

### Open-Weight and SLMs

In the SLM substudy, accuracy spanned roughly 35 pp within a single panel: from 82.22% (407/495) in GPT-OSS 120B (SD 0.55 pp) down to 47.47% (235/495) in Gemma 3 4B (SD 0 pp; Table 7 and Figure 2B). The internal ranking tracked capacity tier broadly but not monotonically: GPT-OSS 120B (465/495, 82.22%) and Llama 4 Scout (390/495, 78.79%) led the open-weight subgroup, followed by Qwen3 8B (359/495, 72.53%) and Gemma 3 27B (323/495, 65.25%); the 3 SLMs clustered lower, with Ministral 3B at 64.04% (317/495) outperforming the larger Gemma 3 27B, Phi-4 at 59.39% (294/495), and Gemma 3 4B at 47.47% (235/495), well above the 20% chance baseline for a 5-alternative MCQ but far below the frontier-panel floor. Between-run SD was ≤3.15 pp in every SLM-panel model, indicating that the 5-run protocol captured residual stochasticity adequately at this accuracy regime as well.

**Table 7:** Exploratory subanalysis: accuracy on Exame Nacional de Avaliação da Formação Médica (ENAMED) 2026 across 7 open-weight and small language models (5 runs × 99 valid items). Kruskal-Wallis H6=33.07; *P*<.001; ε2=0.97; Fleiss κ=0.508; Krippendorff α=.509.

Model	Mean (SD)^a^	95% CI	Correct/total, n/N
GPT-OSS 120B	82.22 (0.55)	81.82–82.63	407/495
Llama 4 Scout	78.79 (1.60)	77.58–80.00	390/495
Qwen3 8B	72.53 (3.15)	70.10–74.95	359/495
Gemma 3 27B	65.25 (1.15)	64.24–66.06	323/495
Ministral 3B	64.04 (1.69)	62.83–65.45	317/495
Phi-4	59.39 (0.45)	58.99–59.60	294/495
Gemma 3 4B	47.47 (0.00)	47.47–47.47	235/495

^a^Mean is expressed in percentage (%), while SD is expressed in percentage points (pp).

Normality and variance homogeneity were rejected for the SLM panel as well (Shapiro-Wilk W=0.92; *P*=.02; n=35; Levene W=3.64; *P*=.009; k=7), motivating the use of Kruskal-Wallis for consistency with the primary analysis. A separate Kruskal-Wallis test on the SLM panel yielded a large effect (H_6_=33.07; *P*<.001; ε^2^=0.97; n=35 runs), consistent with the wide between-model spread. Intermodel agreement was substantially lower than in the frontier panel (Fleiss κ=0.508; Krippendorff α=.509), and 17 items met the default CE criterion under the same ≥3 models × 5-of-5 runs threshold, almost twice the number seen in the frontier panel (Table 8). Importantly, the strongest SLM-panel model (GPT-OSS 120B; 407/495, 82.22%) still ranked below every frontier-panel model except GPT-4o-mini (365/495, 73.74%), and the gap between the best SLM-panel model and the top frontier-panel cluster (Charcot: 480/495, 96.97%; GPT-5: 467/495, 94.34%; Gemini 2.5 Pro: 465/495, 93.94%) was in the 12-15 pp range. Together, the SLM panel quantifies a frontier × subfrontier accuracy gap and a larger error idiosyncrasy (frontier panel κ=0.852 vs SLM κ=0.508) in Portuguese-language board-level medical reasoning.

**Table 8:** Convergence error (CE) items in the small language model (SLM) panel at the default threshold (≥3 models × 5 of 5 runs). Item 77 converged on alternative B across all 7 SLM-panel models, paralleling the 10-of-10 convergence observed in the frontier panel and the subsequent Instituto Nacional de Estudos e Pesquisas Educacionais Anísio Teixeira (INEP) rectification (D to B). A total of 5 of the 17 SLM CE items overlap with the frontier-panel CE set (Q33, Q48, Q77, Q83, and Q97), indicating partially shared failure modes between the 2 panels; the remaining 12 items are specific to the SLM panel and reflect the greater error idiosyncrasy of open-weight and SLMs.

Item	Preliminary key	Convergent wrong	Models, n/N
77	D	B	7/7
9	A	B	6/7
48	D	C	6/7
83	B	C	6/7
35	D	B	5/7
97	C	D	5/7
13	C	A	4/7
38	D	C	4/7
44	B	A	4/7
47	C	A	4/7
57	D	A	4/7
91	B	A	4/7
16	B	A	3/7
29	A	B	3/7
33	C	A	3/7
42	D	A	3/7
88	D	A	3/7

## Discussion

### Principal Findings

In this ENAMED 2026 benchmark, 9 of 10 frontier-panel models exceeded 85% mean accuracy and the Brazilian Portuguese domain-specialized system (Charcot) ranked first at 96.97% (480/495; 95% CI 96.36%-97.58%). Between-model differences were statistically significant (Kruskal-Wallis H_9_=47.65; *P*<.001; ε^2^=0.97) and the ranking was preserved in the item- and run-conditioned GLMM sensitivity analysis. Pairwise post hoc testing distinguished Charcot from the subfrontier cluster (GPT-4o-mini, DeepSeek v3.2-exp, and Grok 4) but not from the top frontier commercial cluster (GPT-5, Gemini 2.5 Pro, Claude Opus 4.1, Claude Sonnet 4.5, GPT-4o, and GPT-4.1). A total of 9 items met the preregistered CE criterion, with item 77 showing 10-of-10 convergence subsequently confirmed by INEP rectification. Intermodel agreement was high in the frontier panel (Fleiss κ=0.852) and substantially lower in the SLM open-weight panel (Fleiss κ=0.508), reflecting greater idiosyncrasy of subfrontier systems.

### Interpretation

Because Charcot’s architecture, training corpus, retrieval components, and inference pipeline are not publicly documented, the performance advantage observed in this study cannot be causally attributed to mechanisms such as Portuguese-language adaptation, domain fine-tuning, retrieval over Brazilian guidelines, prompt engineering, or any combination thereof. Our findings are consistent with prior evidence that domain and language adaptation may improve clinical reasoning in country-specific contexts [6-9,18]. Additionally, it is important to highlight that this study does not constitute mechanistic evidence for specialization but provides black-box comparative evidence under a standardized Portuguese-language protocol. The statistically honest reading is that Charcot is indistinguishable from the top frontier commercial cluster after Holm correction and clearly separable from subfrontier commercial and all open-weight models tested.

The CE construct proved stable across threshold choices (range, 5 to 20 items across a 3 × 3 sensitivity grid) and, in one prospectively flagged case (item 77), anticipated an official INEP rectification. This suggests that CE can serve as a practical item-level signal for examination quality assurance, providing a cheap descriptive screen that surfaces candidates for human review rather than a replacement for expert adjudication. Also, the intermodel agreement metrics (Fleiss κ and Krippendorff α) are complementary to CE, as they summarize the global convergence landscape symmetrically with respect to correctness, whereas CE isolates the asymmetric subset in which convergence occurs on an incorrect alternative.

The positive Spearman correlation between NMRT and accuracy in the 8 retained commercial models (ρ=0.74; *P*=.04) sits close to the critical value for n=8 and depends on 2 prespecified exclusions (Charcot and Grok 4). For this reason, and because NMRT captures end-to-end orchestration latency rather than inference cost, we interpret this association as descriptive of orchestration behavior under the included models rather than as evidence of a general latency–accuracy relationship. It is primarily an artifact of reasoning-native systems (GPT-5 and Gemini 2.5 Pro) occupying both the upper latency and upper accuracy tails of the OpenRouter-routed panel, and it does not survive reinclusion of Grok 4, whose atypical xAI-via-OpenRouter queueing tail compresses the rank statistic.

### Comparison With Prior Work

Prior Brazilian benchmarks of generalist LLMs on the Revalida and on the Brazilian Society of Cardiology certification exam have reported inconsistent performance, attributed partly to language and local clinical context [6-9]. Our results on ENAMED 2026 extend that evidence to a broader generalist curriculum and to a domain-specialized comparator, and add: (1) effect-size reporting alongside omnibus and pairwise tests, (2) a GLMM sensitivity analysis conditioning on item difficulty and run, (3) prespecified intermodel agreement metrics (Fleiss κ and Krippendorff α), and (4) the CE construct, whose prospective flagging of item 77 we interpret as a positive but anecdotal signal of practical utility.

### Methodological Considerations

A unified client layer (OpenRouter) was used for all commercial models. For first-party proprietary models, this is methodologically equivalent to a standardized client library: OpenRouter forwards requests to the canonical provider end points and exposes the same model weights, inference infrastructure, and version pins reported by each provider, while equalizing client-side serialization, retry/timeout handling, and response parsing. Framing OpenRouter as a client-layer control (rather than as a third-party inference rehosting) is supported by documentation and log-level evidence retained in Multimedia Appendix 2. Wall-clock latency measured in this study therefore includes an OpenRouter routing overhead that is systematic across all routed models; relative intermodel NMRT comparisons remain internally valid, whereas absolute NMRT should not be compared with studies using direct provider software development kits.

Temperature=0.0 reduces variability but does not guarantee full determinism [12-14]. The 5-run protocol was chosen as an empirical characterization of residual stochasticity rather than a guarantee of determinism; within-model SD stayed ≤1.53 pp for every model and was 0.00-0.71 pp for 7 of 10 models, supporting the adequacy of 5 runs at this accuracy regime. Reasoning-oriented prompting (eg, chain-of-thought, CoT) was not applied in the primary protocol because (1) reasoning-native frontier models perform internal reasoning by default, making explicit chain-of-thought (CoT) [19] redundant or detrimental to consistency, (2) CoT is known to amplify positional and base-rate biases and to produce post hoc rationalizations in MCQ tasks; and (3) applying CoT selectively to a subset of models would break the standardized-prompt comparability that is the core control of this benchmark. A fully factorial model × prompt-strategy evaluation is outside the scope of this study [20-22].

### Open-Weight and SLMs

The SLM substudy extends the frontier-panel picture to a disjoint capacity range (3B to 120B parameters, open weights) under identical prompting and scoring. Three observations follow. First, the strongest open-weight system tested (GPT-OSS 120B; 407/495, 82.22%) was statistically and substantively below every frontier-panel frontier commercial model except GPT-4o-mini, establishing a frontier × subfrontier accuracy gap of roughly 12-15 pp against the top cluster (Charcot, GPT-5, and Gemini 2.5 Pro). Second, this gap widens sharply at the SLM tier: Ministral 3B, Phi-4, and Gemma 3 4B, none of which exceeded 65% (eg, maximum of 323 out of 495), fell approximately 30-50 pp below Charcot. Third, error patterns in the SLM panel were markedly more idiosyncratic than in the frontier panel (Fleiss κ dropping from 0.852 to 0.508, and the number of CE items nearly doubling, 9 to 17), which is consistent with lower-capacity systems converging on different, less stable wrong alternatives rather than sharing blind spots with each other.

Practically, this has 2 implications: open-weight and SLMs are attractive for deployment under data sovereignty, cost, or offline constraints, but at ENAMED 2026 board-level difficulty in Portuguese they remain below both the Brazilian Portuguese domain-specialized system and the top frontier commercial cluster. Their larger error idiosyncrasy also makes ensemble-based audit signals such as CE noisier in this range. Reducing the frontier × subfrontier gap in Portuguese medical reasoning likely requires either scaling, targeted domain and language adaptation of open-weight systems, or a combination. This result is descriptive and specific to ENAMED 2026; generalization to other Brazilian examinations or to deployment-grade clinical tasks requires further benchmarking.

### Implications for Educational Assessment

The results support cautious use of multimodel ensembles as an audit signal for item banks and answer keys, particularly when consensus conflicts with the official key, as in item 77 of ENAMED 2026. Model consensus should be treated as a cue for expert review rather than as an arbiter of correctness, and CE should be framed as a descriptive screen, not as an inferential test. Beyond item review, the high-accuracy frontier and specialized models demonstrated here may be useful as curated study aids and as components of simulated assessment environments, subject to the usual caveats about MCQ performance does not generalize to clinical communication or decision-making.

### Study Limitations

This study has important limitations. First, it is based on a single examination administered in a single year. Although ENAMED 2026 covers a broad range of topics, it cannot represent all specialties, question formats, or longitudinal shifts in the medical curriculum. Therefore, these results may not generalize to other national examinations or to future ENAMED editions.

Second, performance was assessed exclusively using MCQs. MCQ performance is only a partial proxy for clinical reasoning and does not capture open-ended clinical communication, multistep decision-making, or empathy. As a result, these scores may overestimate real-world clinical capability.

Third, the internal architecture and training characteristics of Charcot are not publicly documented because it is a proprietary model. Its architecture, training corpus, retrieval components, and inference pipeline are therefore not fully transparent. Consequently, the performance differences observed here cannot be attributed specifically to language adaptation, domain fine-tuning, retrieval augmentation, or prompt design.

Another important limitation relates to the OpenRouter routing layer. Accessing models through OpenRouter introduces an additional network hop compared with direct provider software development kits. While this does not affect accuracy, it adds systematic latency overhead. The unified client layer was used to minimize implementation variance across providers, but absolute NMRT values should not be compared directly with studies that used provider-native APIs.

Decoding-level nondeterminism should also be considered. Although temperature was set to 0.0, some output variability remains possible. The 5-run protocol was designed to characterize this residual stochasticity empirically. Additional runs would likely narrow CIs modestly but would be unlikely to substantially alter the overall ranking of models [23].

A further limitation concerns latency analysis. Grok 4 was excluded from NMRT analyses because of an extreme long-tail latency distribution when accessed via xAI through OpenRouter. This exclusion was prespecified and documented, but it means that NMRT comparisons are valid only for the 8 more homogeneously routed commercial models included in that analysis.

In addition, all experiments were conducted from a single orchestrator host in a single geographical region. Results may differ under alternative client-side infrastructure, network paths to provider regions, or rate-limit conditions.

Finally, rationale analysis was descriptive rather than formally validated. The qualitative review of model justifications did not use a formal faithfulness metric. Therefore, findings related to reasoning patterns should be interpreted as illustrative and hypothesis-generating rather than definitive.

### Conclusion

A Brazilian Portuguese domain-specialized system achieved the highest rank on ENAMED 2026, proving statistically indistinguishable from the top frontier commercial cluster. While the lack of architectural disclosure means these results serve as black-box comparative evidence rather than mechanistic proof of specialization, the system’s performance remained significantly above subfrontier and open-weight models. The stability of the CE criterion, which accurately flagged a rectified item (Q77), further supports its adoption as a robust tool for examination quality assurance.

## Data Availability

All datasets (Exame Nacional de Avaliação da Formação Médica [ENAMED] 2026 items, the official Instituto Nacional de Estudos e Pesquisas Educacionais Anísio Teixeira [INEP] answer keys, the OpenRouter provider logs, and the raw JSON outputs from all model runs) are provided in Multimedia Appendix 2, and the analysis code is openly available in the repository referenced in Multimedia Appendix 1. No access restrictions apply to these datasets; the proprietary nature of the Charcot model concerns its architecture and is disclosed in the Conflicts of Interest and Study Limitations sections.

## Appendix

Multimedia Appendix 1 The OpenRouter model identifiers, underlying provider versions, access windows, and inference parameters used for each model in the ENAMED 2026 benchmark.

Multimedia Appendix 2 Complete raw data and reproducibility artefacts for the ENAMED 2026 benchmark study. All artefacts are hosted in a persistent, read-only shared repository.

Multimedia Appendix 3 Additional statistical outputs: main panel.

Multimedia Appendix 4 Additional outputs: exploratory open-weight and small language model substudy.

## Abbreviations

API: application programming interface
CE: convergence error
CoT: chain-of-thought
ENAMED: Exame Nacional de Avaliação da Formação Médica
FDR: false discovery rate
GLMM: generalized linear mixed model
INEP: Instituto Nacional de Estudos e Pesquisas Educacionais Anísio Teixeira
LLM: large language model
MCQ: multiple-choice question
NMRT: normalized mean response time
OR: odds ratio
pp: percentage points
SLM: small language model
