# Supplementary material for: Performance of Large Language Models on the Brazilian National Medical Education Examination: Comparative Benchmark Study
Source: JMIR Med Educ. 2026 May 29;12:e89839. doi: 10.2196/89839 (PMC13263655; doi:10.2196/89839)
Supplement: Multimedia Appendix 1 [file mededu_v12i1e89839_app1.docx]

# Multimedia Appendix 2

## Model Access Metadata

This appendix lists the OpenRouter model identifiers, underlying provider versions, access windows, and inference parameters used for each model in the ENAMED 2026 benchmark.

| Model (display) | Provider | Temperature | Top-p |
| --- | --- | --- | --- |
| Charcot | Voa Health direct | 0.0 | N/A |
| GPT-5 | OpenAI | 0.0 | 1.0 |
| Claude Opus 4.1 | Anthropic | 0.0 | 1.0 |
| Claude Sonnet 4.5 | Anthropic | 0.0 | 1.0 |
| Gemini 2.5 Pro | Google | 0.0 | 1.0 |
| Grok 4 | xAI | 0.0 | 1.0 |
| GPT-4.1 | OpenAI | 0.0 | 1.0 |
| GPT-4o | OpenAI | 0.0 | 1.0 |
| GPT-4o-mini | OpenAI | 0.0 | 1.0 |
| DeepSeek v3.2-exp | DeepSeek | 0.0 | 1.0 |
| GPT-OSS 120B | OpenAI (open-weight) | 0.0 | 1.0 |
| Llama 4 Scout | Meta (open-weight) | 0.0 | 1.0 |
| Qwen3 8B | Alibaba (open-weight) | 0.0 | 1.0 |
| Gemma 3 27B | Google (open-weight) | 0.0 | 1.0 |
| Ministral 3B | Mistral (open-weight) | 0.0 | 1.0 |
| Phi-4 | Microsoft (open-weight) | 0.0 | 1.0 |
| Gemma 3 4B | Google (open-weight) | 0.0 | 1.0 |
|  |  |  |  |

*Table S1. OpenRouter model identifiers and inference parameters*

## A. Prompt Templates

Two linguistically parallel prompt templates were prepared for the study. The Portuguese version was used for all models in the primary protocol; the English translation is provided for reference only and was not used during inference.

**Portuguese prompt (used):**

*Você irá responder a uma prova de assuntos médicos. Analise a questão a seguir e retorne a alternativa correta (A, B, C ou D) com uma breve explicação (reasoning) sobre o motivo da escolha da alternativa correta. Responda obrigatoriamente com a estrutura abaixo:
- answer: A|B|C|D
- reasoning: breve explicação*

**English prompt (translation, not used):**

*You will be answering a medical subject exam. Analyze the following question and return the correct alternative (A, B, C, or D) with a brief explanation (reasoning) for choosing the correct alternative. Answer must use the structure below:
- answer: A|B|C|D
- reasoning: brief explanation*

Note: The prompt was concatenated with the full item text (stem + alternatives) for each question. No additional system prompt, chain-of-thought instruction, or few-shot examples were provided in the primary protocol.

## B. Representative ENAMED 2026 Sample Items

Five items are reproduced below, one per macro-domain covered by ENAMED 2026, to illustrate question format, clinical complexity, and the range of specialties assessed. Items are transcribed verbatim from the official Caderno 01 (exam type 1). The correct alternative according to the preliminary official answer key is indicated after each item.

### Item 1 — Internal Medicine (Clínica Médica)

Mulher de 58 anos, com diagnóstico de hipertensão arterial sistêmica (HAS) e em tratamento irregular, é encaminhada ao ambulatório de clínica médica de atenção secundária. Queixa-se de fadiga e dispneia aos esforços, com piora progressiva.

Ao exame físico, é observado ritmo cardíaco regular em 4 tempos (B3 + B4), sem sopros no precórdio, mas com crépitos em bases pulmonares; pressão arterial: 148 × 90 mmHg.

Ecocardiograma transtorácico evidencia hipertrofia ventricular esquerda concêntrica, associada com fração de ejeção de 38% (por Simpson). Exames laboratoriais normais, salvo pela elevação sérica de peptídeo natriurético tipo B (BNP). Para melhorar o controle da HAS e o prognóstico da paciente, o tratamento com inibidor da enzima conversora de angiotensina foi mantido, e o especialista optou por associar determinado fármaco, devido ao impacto positivo no prognóstico de sobrevida dessa paciente.

O fármaco introduzido no tratamento da paciente foi
(A) espironolactona.
(B) clortalidona.
(C) hidralazina.
(D) clonidina.

**Official answer: A**

### Item 9 — Paediatrics (Pediatria)

Paciente de 7 anos, na 4ª semana de tratamento quimioterápico para leucemia linfocítica aguda, é admitido em hospital terciário com história de febre há 12 horas (temperatura axilar de 39 °C), tosse e dispneia. Após 24 horas da admissão, segue febril, apesar de ter usado dipirona há 1 hora, e mantém diurese de 2 mL/kg/h.

Exame físico: hidratado; pálido; orientado no tempo e no espaço; frequência cardíaca de 130 bpm; temperatura axilar de 39 °C; frequência respiratória de 36 irpm; pressão arterial de 90 × 60 mmHg; boa perfusão periférica; murmúrio vesicular diminuído em base de hemitórax à direita, com crepitações; bulhas taquicárdicas; fígado a 4 cm do rebordo costal direito e 5 cm do apêndice xifoide; baço a 4 cm do rebordo costal esquerdo.

Os exames laboratoriais evidenciam lactato aumentado, acidose metabólica e hemograma com leucocitose com desvio à esquerda.

Com base no quadro descrito, o diagnóstico é
(A) sepse.
(B) choque séptico.
(C) disfunção de múltiplos órgãos.
(D) síndrome da resposta inflamatória sistêmica.

**Official answer: A**

### Item 12 — Primary Care / Public Health (Saúde Coletiva / Atenção Primária)

Homem de 52 anos, branco, solteiro, comparece à consulta agendada na Unidade Básica de Saúde (UBS) desejando realizar revisão clínica e exames laboratoriais. Desde os 35 anos não faz acompanhamento de saúde. Relata história familiar de diabetes e hipertensão, e a mãe faleceu com câncer de pulmão. Sem história familiar de câncer de próstata. Fuma cerca de 2 maços por dia há 21 anos. Exame físico: pressão arterial de 120 × 80 mmHg, índice de massa corporal de 23 kg/m², sem outras alterações.

Considerando as recomendações de rastreamento para esse paciente, o médico de família e comunidade deve

(A) solicitar exames de colesterol total e frações, hemograma, glicemia de jejum, creatinina, PSA, radiografia de tórax, colonoscopia, realizar toque retal; orientar sobre a prática de atividade física regular.
(B) solicitar exames de colesterol total, glicemia de jejum, pesquisa de sangue oculto nas fezes, PSA, ofertar anti-HIV e HBsAg, realizar toque retal; orientar sobre participação no grupo na UBS para abandono do tabagismo.
(C) abordar mudanças no estilo de vida e cessação do tabagismo; acompanhar, em consultas longitudinais, as futuras possibilidades de exames complementares, quando o paciente atingir faixa etária para investigações adicionais.
(D) solicitar exames de colesterol total, HDL e triglicerídeos, glicemia de jejum, pesquisa de sangue oculto nas fezes, ofertar testes rápidos para HIV, sífilis e hepatites B e C; realizar abordagem sobre possibilidade de cessação do tabagismo.

**Official answer: D**

### Item 17 — Surgery (Cirurgia)

Paciente de 20 anos, sexo masculino, vítima de colisão "automóvel a muro", sem cinto de segurança, é atendido ainda na cena pelo Serviço Móvel de Atendimento de Urgência (SAMU). Exame físico: paciente torporoso; saturação de O₂ de 60%, em ar ambiente; frequência respiratória de 28 irpm; frequência cardíaca de 112 bpm; pressão arterial de 90 × 50 mmHg. Desvio da traqueia para a direita, turgência de veias jugulares, hipofonese de bulhas cardíacas e diminuição acentuada do murmúrio vesicular à esquerda.

Qual é a conduta adequada no atendimento pré-hospitalar?
(A) Reposição volêmica.
(B) Cricotireoidostomia.
(C) Pericardiocentese.
(D) Toracocentese.

**Official answer: D**

### Item 32 — Obstetrics / Gynaecology (Ginecologia e Obstetrícia)

Paciente G5P3C1, 35 anos, idade gestacional de 15 semanas por ecografia realizada com 8 semanas, hipertensa crônica em uso de enalapril, antecedente de pré-eclâmpsia. Comparece à consulta de pré-natal na Unidade Básica de Saúde (UBS) com pressão arterial de 140 × 90 mmHg.

Qual é a conduta medicamentosa indicada para essa paciente?
(A) Captopril, varfarina e ácido acetilsalicílico.
(B) Furosemida, varfarina e carbonato de cálcio.
(C) Losartana, enoxaparina e carbonato de cálcio.
(D) Alfa-metildopa, ácido acetilsalicílico e carbonato de cálcio.

**Official answer: D**
