# Supplementary material for: Performance of Large Language Models on the Brazilian National Medical Education Examination: Comparative Benchmark Study
Source: JMIR Med Educ. 2026 May 29;12:e89839. doi: 10.2196/89839 (PMC13263655; doi:10.2196/89839)
Supplement: Multimedia Appendix 2 [file mededu_v12i1e89839_app2.docx]

# Multimedia Appendix 1

## Raw Data and Reproducibility Artefacts

This appendix provides access to the complete raw data and reproducibility artefacts for the ENAMED 2026 benchmark study. Due to file-size constraints inherent to journal supplementary material, all artefacts are hosted in a persistent, read-only shared repository.

## Data Repository Link

**https://drive.google.com/drive/folders/13AvfmOp1LVdpddpuFDcSIYUgWX6kk_B_?usp=sharing**

## Repository Contents

**results/** Raw JSON outputs from all 17 models × 5 runs (85 files). Each file contains the model's responses, selected alternatives, rationale text, and wall-clock elapsed time per item.

**answer_keys/** Official ENAMED 2026 preliminary answer key (Caderno 01, type 1) and the post-rectification key (item 77: D → B).
