# Supplementary material for: Performance of Large Language Models on the Brazilian National Medical Education Examination: Comparative Benchmark Study
Source: JMIR Med Educ. 2026 May 29;12:e89839. doi: 10.2196/89839 (PMC13263655; doi:10.2196/89839)
Supplement: Multimedia Appendix 3 [file mededu_v12i1e89839_app3.docx]

# Multimedia Appendix 3

## Supplementary Statistical Outputs: Main Panel

## S3.1 Normality and Variance Homogeneity

| Test | W / statistic | P | n | k |
| --- | --- | --- | --- | --- |
| Shapiro Wilk | 0.8190 | < .001 | 50 | — |
| Levene | 1.3048 | .265 | — | 10 |

*Table S4. Normality (Shapiro–Wilk) and variance homogeneity (Levene) tests on per-run accuracy (main panel, n = 50, k = 10).*

## S3.2 Kruskal–Wallis Test

| H | P | df | k | n | ε² | η²_H |
| --- | --- | --- | --- | --- | --- | --- |
| 47.66 | < .001 | 9 | 10 | 50 | 0.9726 | 0.9665 |

*Table S5. Kruskal–Wallis omnibus test (main panel).*

## S3.3 Full Dunn–Holm Pairwise Comparisons

| Model A | Model B | P (Holm) | \|r\| | Sig. |
| --- | --- | --- | --- | --- |
| Charcot | GPT-4o-mini | < .001 | 1.00 | Yes |
| Charcot | DeepSeek v3.2-exp | < .001 | 1.00 | Yes |
| GPT-4o-mini | GPT-5 | .002 | 1.00 | Yes |
| Gemini 2.5 Pro | GPT-4o-mini | .003 | 1.00 | Yes |
| Charcot | Grok 4 | .006 | 1.00 | Yes |
| DeepSeek v3.2-exp | GPT-5 | .014 | 1.00 | Yes |
| DeepSeek v3.2-exp | Gemini 2.5 Pro | .021 | 1.00 | Yes |
| Claude Opus 4.1 | GPT-4o-mini | .049 | 1.00 | Yes |
| Charcot | GPT-4.1 | .053 | 1.00 | No |
| GPT-5 | Grok 4 | .094 | 1.00 | No |
| Gemini 2.5 Pro | Grok 4 | .130 | 1.00 | No |
| Claude Opus 4.1 | DeepSeek v3.2-exp | .256 | 1.00 | No |
| Claude Sonnet 4.5 | GPT-4o-mini | .256 | 1.00 | No |
| Charcot | GPT-4o | .292 | 1.00 | No |
| GPT-4.1 | GPT-5 | .508 | 1.00 | No |
| Gemini 2.5 Pro | GPT-4.1 | .658 | 1.00 | No |
| GPT-4o | GPT-4o-mini | .658 | 1.00 | No |
| Claude Sonnet 4.5 | Charcot | .709 | 1.00 | No |
| Claude Opus 4.1 | Grok 4 | .926 | 1.00 | No |
| Claude Sonnet 4.5 | DeepSeek v3.2-exp | .926 | 1.00 | No |
| Claude Sonnet 4.5 | GPT-5 | 1.000 | 0.92 | No |
| Claude Sonnet 4.5 | GPT-4o | 1.000 | 0.40 | No |
| Claude Opus 4.1 | GPT-4o | 1.000 | 1.00 | No |
| Claude Opus 4.1 | GPT-5 | 1.000 | 0.80 | No |
| Charcot | GPT-5 | 1.000 | 0.96 | No |
| Charcot | Gemini 2.5 Pro | 1.000 | 1.00 | No |
| Claude Sonnet 4.5 | GPT-4.1 | 1.000 | 0.88 | No |
| Claude Sonnet 4.5 | Gemini 2.5 Pro | 1.000 | 1.00 | No |
| Claude Opus 4.1 | Gemini 2.5 Pro | 1.000 | 1.00 | No |
| Claude Opus 4.1 | GPT-4.1 | 1.000 | 1.00 | No |
| Claude Opus 4.1 | Charcot | 1.000 | 1.00 | No |
| Claude Opus 4.1 | Claude Sonnet 4.5 | 1.000 | 0.60 | No |
| Gemini 2.5 Pro | GPT-4o | 1.000 | 1.00 | No |
| DeepSeek v3.2-exp | Grok 4 | 1.000 | 1.00 | No |
| DeepSeek v3.2-exp | GPT-4o | 1.000 | 1.00 | No |
| DeepSeek v3.2-exp | GPT-4o-mini | 1.000 | 1.00 | No |
| DeepSeek v3.2-exp | GPT-4.1 | 1.000 | 1.00 | No |
| Claude Sonnet 4.5 | Grok 4 | 1.000 | 1.00 | No |
| GPT-4.1 | GPT-4o | 1.000 | 0.80 | No |
| Gemini 2.5 Pro | GPT-5 | 1.000 | 0.32 | No |
| GPT-4.1 | Grok 4 | 1.000 | 0.96 | No |
| GPT-4.1 | GPT-4o-mini | 1.000 | 1.00 | No |
| GPT-4o | Grok 4 | 1.000 | 1.00 | No |
| GPT-4o | GPT-5 | 1.000 | 1.00 | No |
| GPT-4o-mini | Grok 4 | 1.000 | 1.00 | No |

*Table S6. All 45 Dunn pairwise comparisons with Holm correction (main panel). |r| = rank-biserial magnitude; direction given by mean ordering in Table 2 of the main text.*

## S3.4 GLMM Coefficient Table

| Comparator vs. Charcot | β | SE | OR | 95% CI |
| --- | --- | --- | --- | --- |
| Claude Opus 4.1 | -1.384 | 0.246 | 0.251 | 0.155–0.406 |
| Claude Sonnet 4.5 | -1.562 | 0.239 | 0.210 | 0.131–0.335 |
| DeepSeek v3.2-exp | -2.876 | 0.198 | 0.056 | 0.038–0.083 |
| Gemini 2.5 Pro | -0.699 | 0.276 | 0.497 | 0.290–0.853 |
| GPT-4.1 | -1.942 | 0.225 | 0.143 | 0.092–0.223 |
| GPT-4o | -1.675 | 0.234 | 0.187 | 0.118–0.296 |
| GPT-4o-mini | -4.934 | 0.186 | 0.007 | 0.005–0.010 |
| GPT-5 | -0.541 | 0.283 | 0.582 | 0.334–1.014 |
| Grok 4 | -2.372 | 0.211 | 0.093 | 0.062–0.141 |

*Table S7. Binomial GLMM coefficients (reference = Charcot). OR < 1 indicates lower odds of a correct response relative to Charcot.*

## S3.5 CE Sensitivity Grid

| Model threshold | Run threshold | N items | Items |
| --- | --- | --- | --- |
| ≥ 2 models | 3 of 5 runs | 20 | 6;9;21;26;33;44;48;66;77;80;83;87;88;91;91;94;96;97;98;100 |
| ≥ 3 models | 3 of 5 runs | 13 | 9;33;44;48;66;77;83;87;91;91;94;97;100 |
| ≥ 4 models | 3 of 5 runs | 7 | 33;48;77;87;94;97;100 |
| ≥ 2 models | 4 of 5 runs | 18 | 6;9;21;26;33;44;48;66;77;80;83;87;91;94;96;97;98;100 |
| ≥ 3 models | 4 of 5 runs | 11 | 9;33;44;48;66;77;83;87;94;97;100 |
| ≥ 4 models | 4 of 5 runs | 6 | 33;48;77;94;97;100 |
| ≥ 2 models | 5 of 5 runs | 18 | 6;9;21;26;33;44;48;66;77;80;83;87;91;94;96;97;98;100 |
| ≥ 3 models | 5 of 5 runs | 9 | 33;48;66;77;83;87;94;97;100 |
| ≥ 4 models | 5 of 5 runs | 5 | 33;48;77;97;100 |

*Table S8. CE sensitivity grid (main panel). Default = ≥ 3 models × 5/5 runs (9 items).*

## S3.6 Inter-model Agreement

| Metric | Value | Items | Raters |
| --- | --- | --- | --- |
| Fleiss Kappa | 0.8516 | 495 | 10 |
| Krippendorff Alpha | 0.8516 | 495 | 10 |

*Table S9. Inter-model agreement (main panel). Fleiss κ and Krippendorff α computed over 99 valid items × 5 runs (495 ratings per model).*

## S3.7 Between-Run Variability

| Model | Runs | Mean (%) | SD (pp) | CV (%) | Range (pp) |
| --- | --- | --- | --- | --- | --- |
| Claude Opus 4.1 | 5 | 91.92 | 0.00 | 0.00 | 0.00 |
| GPT-4o | 5 | 90.91 | 0.00 | 0.00 | 0.00 |
| Grok 4 | 5 | 88.08 | 0.45 | 0.51 | 101.01 |
| Claude Sonnet 4.5 | 5 | 91.31 | 0.55 | 0.61 | 101.01 |
| Charcot | 5 | 96.97 | 0.71 | 0.74 | 202.02 |
| Gemini 2.5 Pro | 5 | 93.94 | 0.71 | 0.76 | 202.02 |
| GPT-4.1 | 5 | 89.90 | 0.71 | 0.79 | 202.02 |
| GPT-4o-mini | 5 | 73.74 | 1.01 | 1.37 | 202.02 |
| DeepSeek v3.2-exp | 5 | 85.66 | 1.11 | 1.29 | 303.03 |
| GPT-5 | 5 | 94.34 | 1.53 | 1.62 | 404.04 |

*Table S10. Between-run variability (main panel).*

## S3.8 NMRT–Accuracy Correlation

| Spearman ρ | P | n |
| --- | --- | --- |
| 0.738 | .037 | 8 |

*Table S11. Spearman correlation between mean NMRT and mean accuracy (main panel, n = 8 retained models; Charcot and Grok 4 excluded).*

## S3.9 Per-Question FDR-Corrected χ² Tests

| Item | χ² | df | P (FDR-BH) | −log₁₀(P) |
| --- | --- | --- | --- | --- |
| Q13 | 50.00 | 9 | < .001 | 6.97 |
| Q20 | 50.00 | 9 | < .001 | 6.97 |
| Q21 | 50.00 | 9 | < .001 | 6.97 |
| Q25 | 50.00 | 9 | < .001 | 6.97 |
| Q39 | 50.00 | 9 | < .001 | 6.97 |
| Q40 | 50.00 | 9 | < .001 | 6.97 |
| Q47 | 50.00 | 9 | < .001 | 6.97 |
| Q52 | 50.00 | 9 | < .001 | 6.97 |
| Q56 | 50.00 | 9 | < .001 | 6.97 |
| Q80 | 50.00 | 9 | < .001 | 6.97 |
| Q87 | 50.00 | 9 | < .001 | 6.97 |
| Q92 | 50.00 | 9 | < .001 | 6.97 |
| Q96 | 50.00 | 9 | < .001 | 6.97 |
| Q98 | 50.00 | 9 | < .001 | 6.97 |
| Q94 | 46.79 | 9 | < .001 | 6.37 |
| Q26 | 46.03 | 9 | < .001 | 6.23 |
| Q44 | 46.03 | 9 | < .001 | 6.23 |
| Q6 | 45.34 | 9 | < .001 | 6.10 |
| Q100 | 45.34 | 9 | < .001 | 6.10 |
| Q83 | 44.65 | 9 | < .001 | 5.97 |
| Q97 | 43.42 | 9 | < .001 | 5.74 |
| Q66 | 42.87 | 9 | < .001 | 5.64 |
| Q16 | 42.42 | 9 | < .001 | 5.56 |
| Q88 | 41.07 | 9 | < .001 | 5.31 |
| Q38 | 39.60 | 9 | < .001 | 5.05 |
| Q12 | 39.13 | 9 | < .001 | 4.96 |
| Q33 | 38.34 | 9 | < .001 | 4.82 |
| Q9 | 37.52 | 9 | < .001 | 4.67 |
| Q91 | 33.96 | 9 | < .001 | 4.04 |
| Q63 | 32.22 | 9 | < .001 | 3.74 |
| Q42 | 28.72 | 9 | .002 | 3.14 |
| Q72 | 28.72 | 9 | .002 | 3.14 |
| Q48 | 23.33 | 9 | .016 | 2.26 |
| Q11 | 22.83 | 9 | .019 | 2.18 |

*Table S12. Per-question χ² tests with Benjamini–Hochberg FDR correction (main panel). Only the 34 items reaching significance are shown.*

# Supplementary Figures — Main Panel


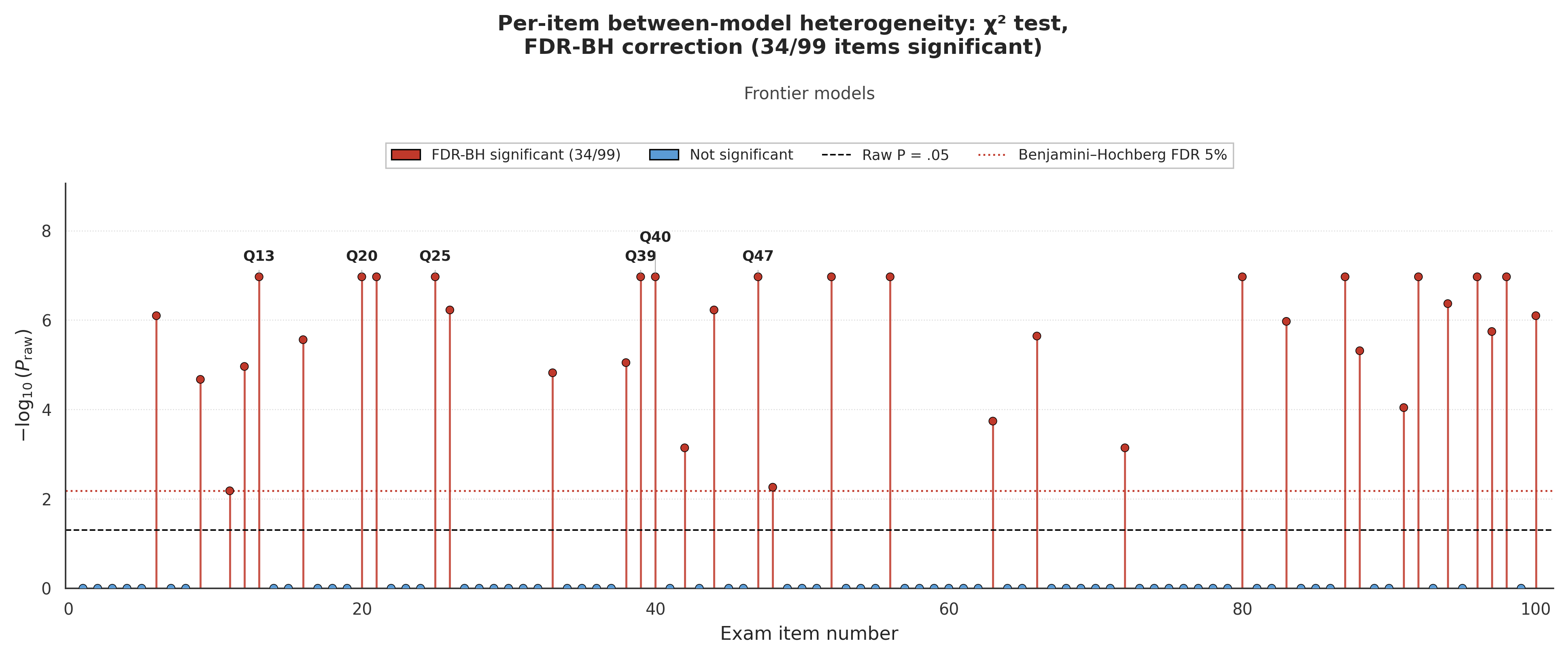


*Figure S1. Per-question FDR-corrected χ² significance scatter (main panel). Each dot is one ENAMED item; the horizontal line marks −log₁₀(0.05).*


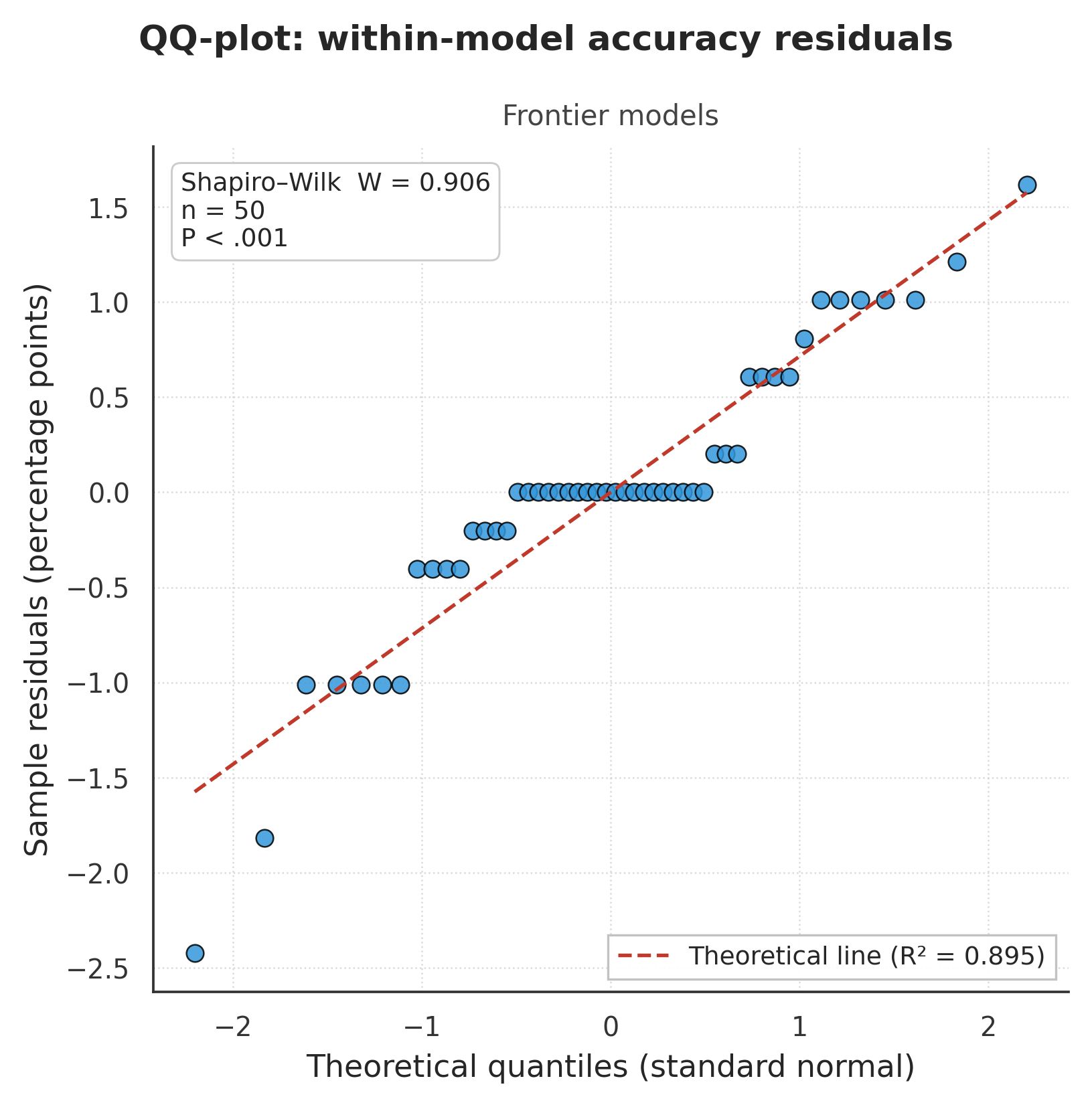


*Figure S2. QQ plot of within-model accuracy residuals (main panel). Shapiro–Wilk on residuals W = 0.906, n = 50, P < .001. This supplements the raw per-run normality test (W = 0.82, P < .001) reported in the main text.*


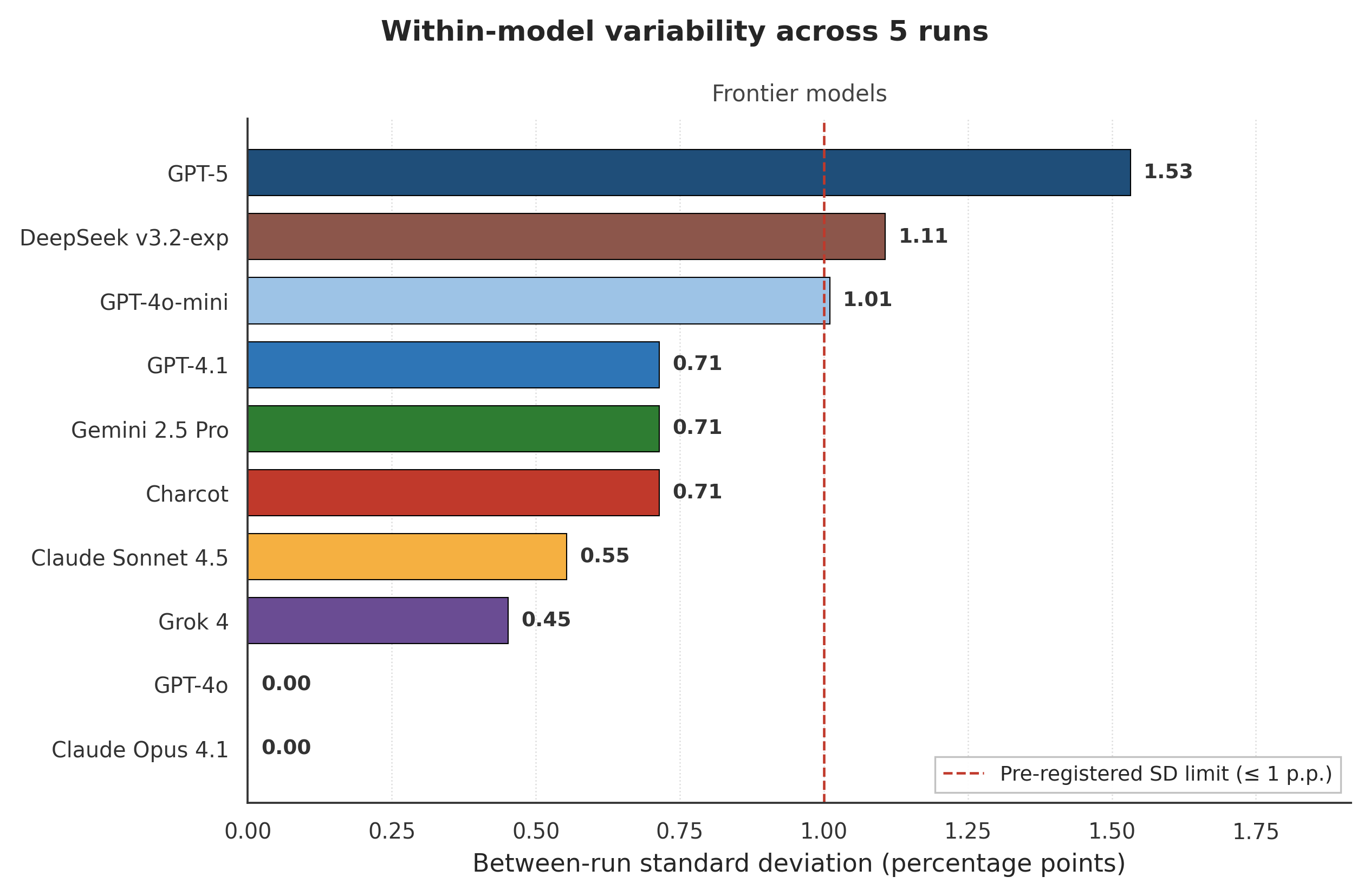


*Figure S3. Between-run variability per model (main panel). Each bar represents the range (pp) across 5 runs.*


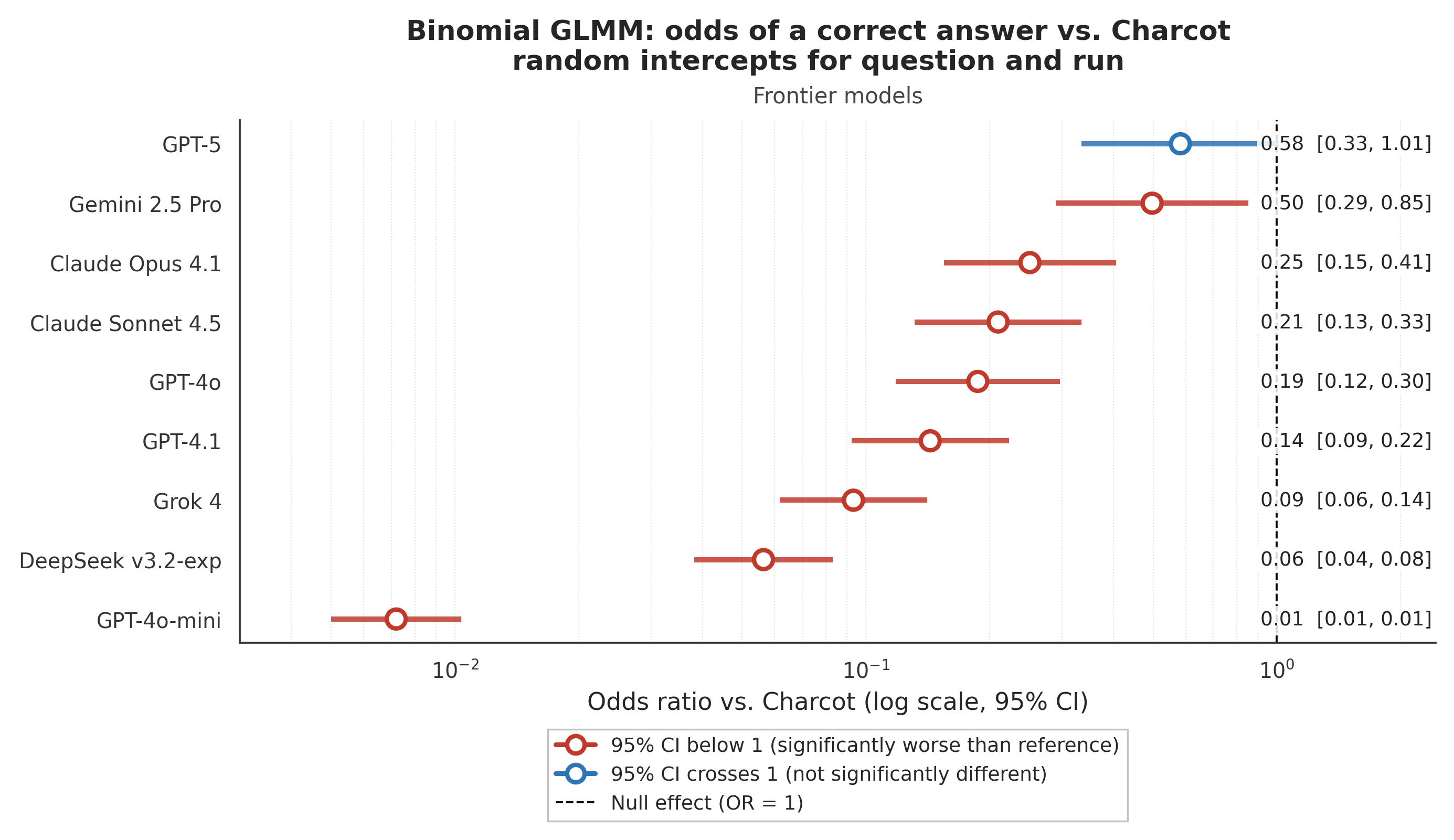


*Figure S4. GLMM forest plot (main panel). Odds ratios with 95% CI relative to Charcot (reference).*
