# Supplementary material for: Performance of Large Language Models on the Brazilian National Medical Education Examination: Comparative Benchmark Study
Source: JMIR Med Educ. 2026 May 29;12:e89839. doi: 10.2196/89839 (PMC13263655; doi:10.2196/89839)
Supplement: Multimedia Appendix 4 [file mededu_v12i1e89839_app4.docx]

# Multimedia Appendix 4

## Supplementary Outputs: Exploratory Open-Weight and Small Language Model Sub-Study

## S4.1 Normality and Variance Homogeneity

| Test | W / statistic | P | n | k |
| --- | --- | --- | --- | --- |
| Shapiro Wilk | 0.9213 | .016 | 35 | — |
| Levene | 3.6354 | .009 | — | 7 |

*Table S13. Normality (Shapiro–Wilk) and variance homogeneity (Levene) tests on per-run accuracy (exploratory panel, n = 35, k = 7).*

## S4.2 Kruskal–Wallis Test

| H | P | df | k | n | ε² | η²_H |
| --- | --- | --- | --- | --- | --- | --- |
| 33.07 | < .001 | 6 | 7 | 35 | 0.9725 | 0.9666 |

*Table S14. Kruskal–Wallis omnibus test (exploratory panel).*

## S4.3 Full Dunn–Holm Pairwise Comparisons

| Model A | Model B | P (Holm) | \|r\| | Sig. |
| --- | --- | --- | --- | --- |
| Gemma 3 4B | GPT-OSS 120B | < .001 | 1.00 | Yes |
| Gemma 3 4B | Llama 4 Scout | .002 | 1.00 | Yes |
| Phi-4 | GPT-OSS 120B | .002 | 1.00 | Yes |
| Gemma 3 4B | Qwen3 8B | .035 | 1.00 | Yes |
| Llama 4 Scout | Phi-4 | .035 | 1.00 | Yes |
| Ministral 3B | GPT-OSS 120B | .061 | 1.00 | No |
| Gemma 3 27B | GPT-OSS 120B | .175 | 1.00 | No |
| Phi-4 | Qwen3 8B | .284 | 1.00 | No |
| Gemma 3 27B | Gemma 3 4B | .442 | 1.00 | No |
| Llama 4 Scout | Ministral 3B | .442 | 1.00 | No |
| Gemma 3 4B | Ministral 3B | .884 | 1.00 | No |
| Gemma 3 27B | Llama 4 Scout | .884 | 1.00 | No |
| Gemma 3 4B | Phi-4 | 1.000 | 1.00 | No |
| Gemma 3 27B | Qwen3 8B | 1.000 | 1.00 | No |
| Gemma 3 27B | Phi-4 | 1.000 | 1.00 | No |
| Gemma 3 27B | Ministral 3B | 1.000 | 0.48 | No |
| Phi-4 | Ministral 3B | 1.000 | 1.00 | No |
| Llama 4 Scout | Qwen3 8B | 1.000 | 1.00 | No |
| Llama 4 Scout | GPT-OSS 120B | 1.000 | 1.00 | No |
| Ministral 3B | Qwen3 8B | 1.000 | 1.00 | No |
| GPT-OSS 120B | Qwen3 8B | 1.000 | 1.00 | No |

*Table S15. All 21 Dunn pairwise comparisons with Holm correction (exploratory panel). |r| = rank-biserial magnitude.*

## S4.4 GLMM Coefficient Table

| Comparator vs. GPT-OSS 120B | β | SE | OR | 95% CI |
| --- | --- | --- | --- | --- |
| Gemma 3 27B | -1.457 | 0.124 | 0.233 | 0.183–0.297 |
| Gemma 3 4B | -2.751 | 0.121 | 0.064 | 0.050–0.081 |
| Llama 4 Scout | -0.296 | 0.141 | 0.744 | 0.564–0.981 |
| Phi-4 | -1.893 | 0.121 | 0.151 | 0.119–0.191 |
| Ministral 3B | -1.549 | 0.123 | 0.212 | 0.167–0.270 |
| Qwen3 8B | -0.873 | 0.131 | 0.418 | 0.323–0.540 |

*Table S16. Binomial GLMM coefficients (reference = GPT-OSS 120B). OR < 1 indicates lower odds of a correct response relative to the reference model.*

## S4.5 CE Sensitivity Grid

| Model threshold | Run threshold | N items | Items |
| --- | --- | --- | --- |
| ≥ 2 models | 3 of 5 runs | 47 | 9;11;13;16;17;17;20;23;25;26;28;29;31;33;35;38;39;42;43;44;47;48;50;56;57;58;60;62;77;80;82;83;86;87;87;88;90;91;92;94;95;96;96;97;98;98;100 |
| ≥ 3 models | 3 of 5 runs | 28 | 9;13;16;20;26;29;31;33;35;38;39;42;43;44;47;48;57;58;77;80;83;86;87;88;91;97;98;100 |
| ≥ 4 models | 3 of 5 runs | 19 | 9;13;20;26;29;33;35;38;39;44;47;48;57;77;80;83;88;91;97 |
| ≥ 2 models | 4 of 5 runs | 41 | 9;11;13;16;17;17;23;26;28;29;31;33;35;38;39;42;43;44;47;48;50;56;57;58;60;77;80;82;83;86;87;88;90;91;94;95;96;97;98;98;100 |
| ≥ 3 models | 4 of 5 runs | 18 | 9;13;16;29;33;35;38;42;43;44;47;48;57;77;83;88;91;97 |
| ≥ 4 models | 4 of 5 runs | 14 | 9;13;29;33;35;38;44;47;48;57;77;83;91;97 |
| ≥ 2 models | 5 of 5 runs | 35 | 9;11;13;16;17;17;26;29;33;35;38;39;42;43;44;47;48;50;56;57;60;77;82;83;86;87;88;90;91;95;96;97;98;98;100 |
| ≥ 3 models | 5 of 5 runs | 17 | 9;13;16;29;33;35;38;42;44;47;48;57;77;83;88;91;97 |
| ≥ 4 models | 5 of 5 runs | 12 | 9;13;35;38;44;47;48;57;77;83;91;97 |

*Table S17. CE sensitivity grid (exploratory panel). Default = ≥ 3 models × 5/5 runs (17 items).*

## S4.6 Inter-model Agreement

| Metric | Value | Items | Raters |
| --- | --- | --- | --- |
| Fleiss Kappa | 0.5085 | 495 | 7 |
| Krippendorff Alpha | 0.5086 | 495 | 7 |

*Table S18. Inter-model agreement (exploratory panel).*

## S4.7 Between-Run Variability

| Model | Runs | Mean (%) | SD (pp) | CV (%) | Range (pp) |
| --- | --- | --- | --- | --- | --- |
| Gemma 3 4B | 5 | 47.47 | 0.00 | 0.00 | 0.00 |
| Phi-4 | 5 | 59.39 | 0.45 | 0.76 | 101.01 |
| GPT-OSS 120B | 5 | 82.22 | 0.55 | 0.67 | 101.01 |
| Gemma 3 27B | 5 | 65.25 | 1.15 | 1.76 | 303.03 |
| Llama 4 Scout | 5 | 78.79 | 1.60 | 2.03 | 404.04 |
| Ministral 3B | 5 | 64.04 | 1.69 | 2.64 | 404.04 |
| Qwen3 8B | 5 | 72.53 | 3.15 | 4.34 | 707.07 |

*Table S19. Between-run variability (exploratory panel).*

## S4.8 Per-Run Accuracy

| Model | Run | Correct | Valid items | Accuracy (%) |
| --- | --- | --- | --- | --- |
| Gemma 3 27B | 1 | 63 | 99 | 63.64 |
| Gemma 3 27B | 2 | 66 | 99 | 66.67 |
| Gemma 3 27B | 3 | 65 | 99 | 65.66 |
| Gemma 3 27B | 4 | 65 | 99 | 65.66 |
| Gemma 3 27B | 5 | 64 | 99 | 64.65 |
| Gemma 3 4B | 1 | 47 | 99 | 47.47 |
| Gemma 3 4B | 2 | 47 | 99 | 47.47 |
| Gemma 3 4B | 3 | 47 | 99 | 47.47 |
| Gemma 3 4B | 4 | 47 | 99 | 47.47 |
| Gemma 3 4B | 5 | 47 | 99 | 47.47 |
| Llama 4 Scout | 1 | 77 | 99 | 77.78 |
| Llama 4 Scout | 2 | 79 | 99 | 79.80 |
| Llama 4 Scout | 3 | 80 | 99 | 80.81 |
| Llama 4 Scout | 4 | 78 | 99 | 78.79 |
| Llama 4 Scout | 5 | 76 | 99 | 76.77 |
| Phi-4 | 1 | 59 | 99 | 59.60 |
| Phi-4 | 2 | 58 | 99 | 58.59 |
| Phi-4 | 3 | 59 | 99 | 59.60 |
| Phi-4 | 4 | 59 | 99 | 59.60 |
| Phi-4 | 5 | 59 | 99 | 59.60 |
| Ministral 3B | 1 | 64 | 99 | 64.65 |
| Ministral 3B | 2 | 66 | 99 | 66.67 |
| Ministral 3B | 3 | 62 | 99 | 62.63 |
| Ministral 3B | 4 | 62 | 99 | 62.63 |
| Ministral 3B | 5 | 63 | 99 | 63.64 |
| GPT-OSS 120B | 1 | 81 | 99 | 81.82 |
| GPT-OSS 120B | 2 | 82 | 99 | 82.83 |
| GPT-OSS 120B | 3 | 82 | 99 | 82.83 |
| GPT-OSS 120B | 4 | 81 | 99 | 81.82 |
| GPT-OSS 120B | 5 | 81 | 99 | 81.82 |
| Qwen3 8B | 1 | 75 | 99 | 75.76 |
| Qwen3 8B | 2 | 70 | 99 | 70.71 |
| Qwen3 8B | 3 | 75 | 99 | 75.76 |
| Qwen3 8B | 4 | 68 | 99 | 68.69 |
| Qwen3 8B | 5 | 71 | 99 | 71.72 |

*Table S20. Per-run accuracy (exploratory panel, 7 models × 5 runs).*

# Supplementary Figures: Exploratory Panel


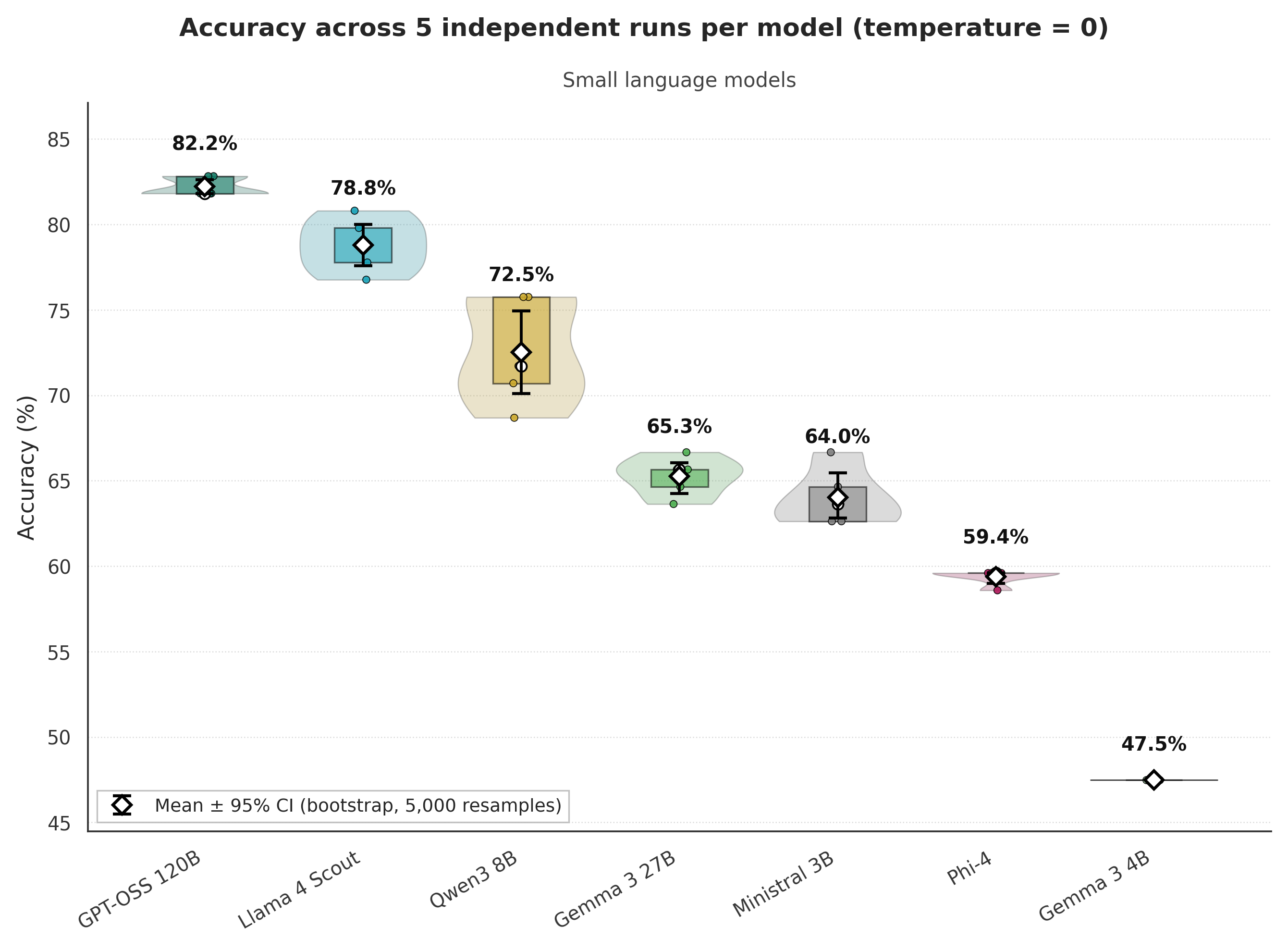


*Figure S5. Accuracy with 95% CI (exploratory panel). Error bars span the bootstrap-free min–max range across 5 runs.*


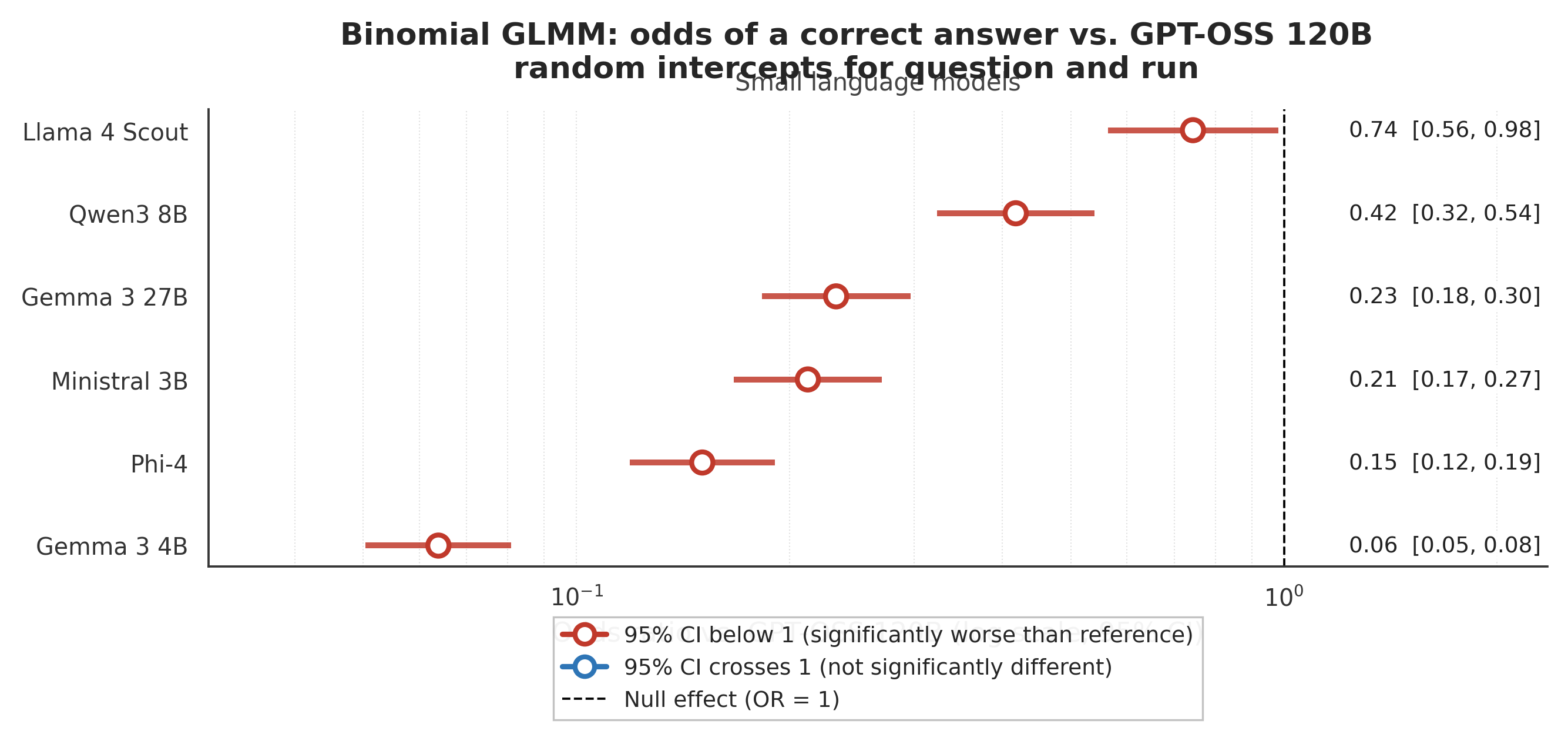


*Figure S6. GLMM forest plot (exploratory panel, reference = GPT-OSS 120B).*


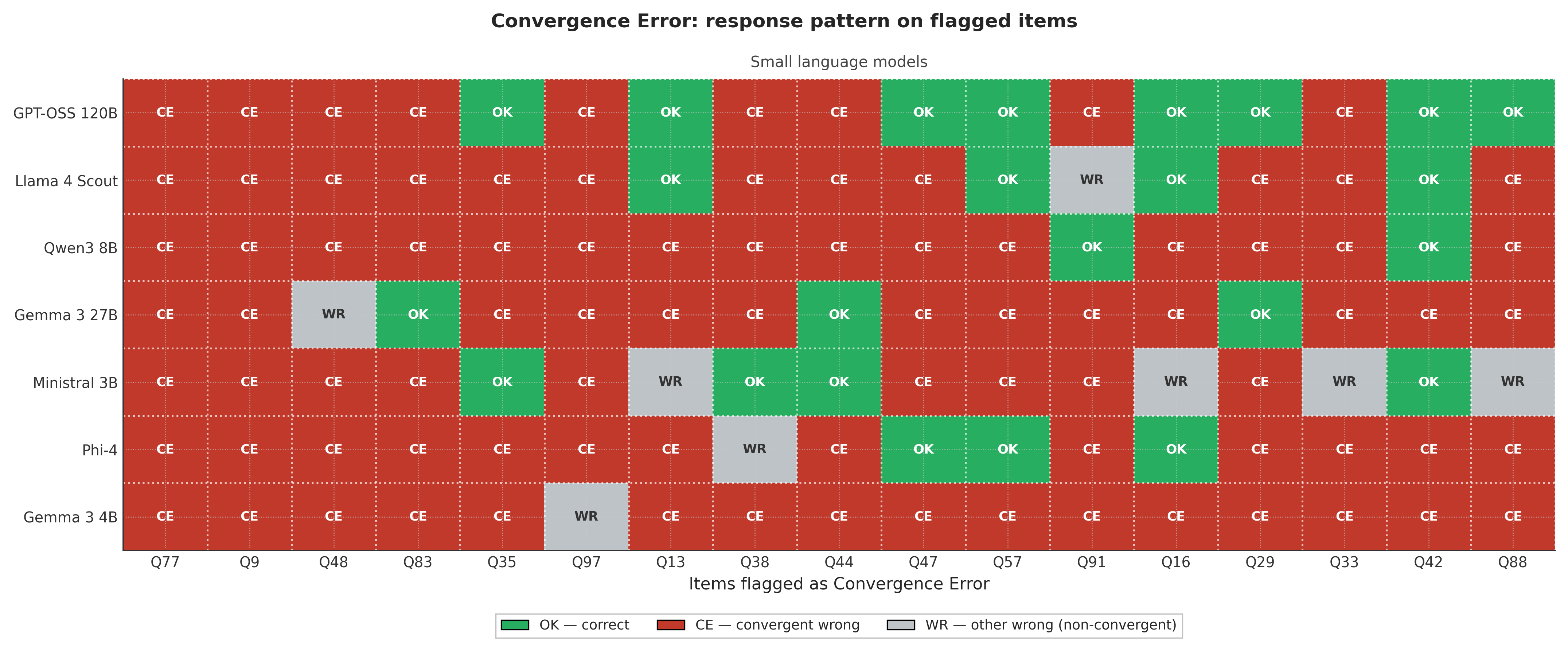


*Figure S7. Convergence Error heatmap (exploratory panel). Rows = models, columns = items meeting the CE criterion.*


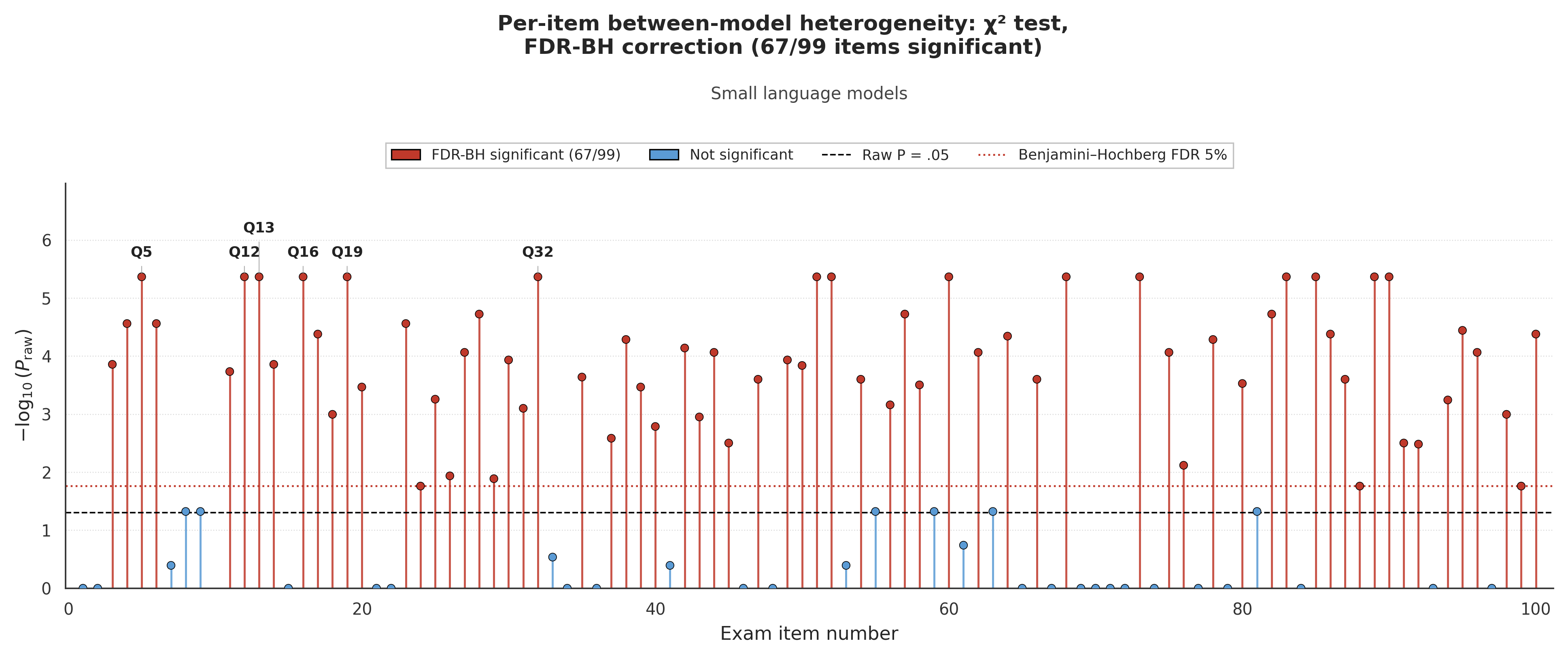


*Figure S8. Per-question FDR-corrected χ² significance scatter (exploratory panel).*


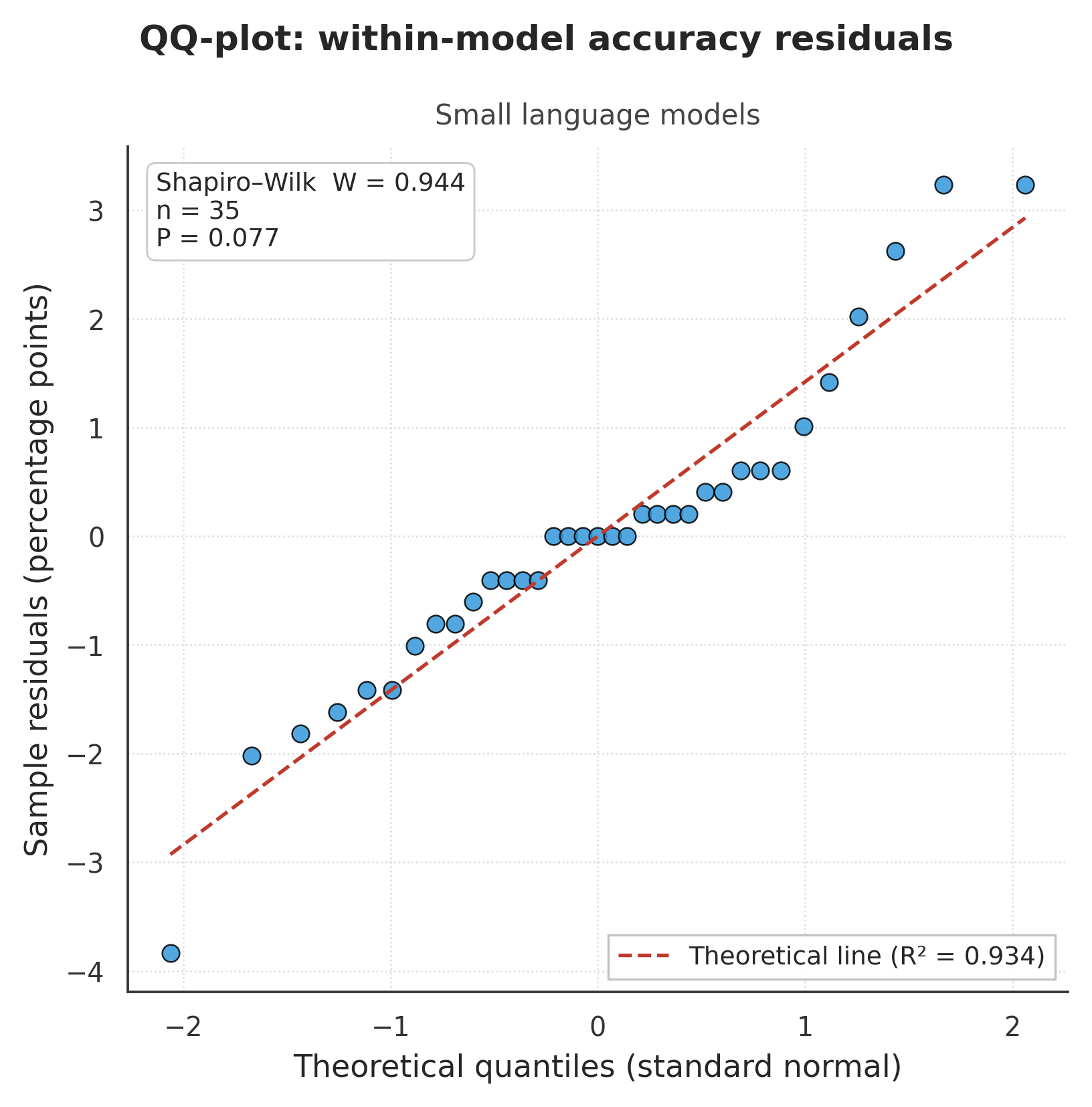


*Figure S9. QQ plot of within-model accuracy residuals (exploratory panel). Shapiro–Wilk on residuals W = 0.944, P = .077, n = 35. This supplements the raw per-run normality test (W = 0.92, P = .02) reported in the main text.*


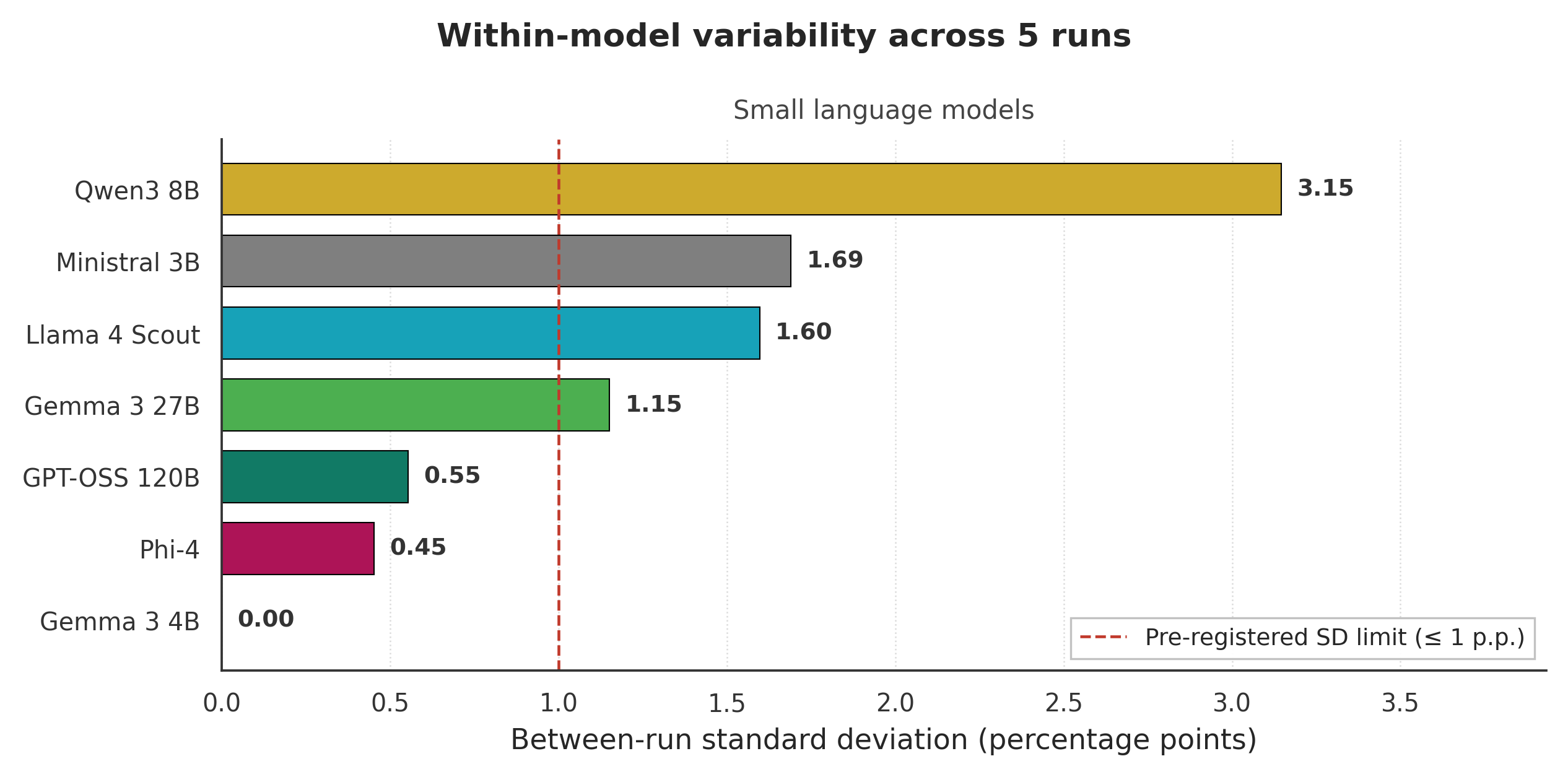


*Figure S10. Between-run variability per model (exploratory panel).*


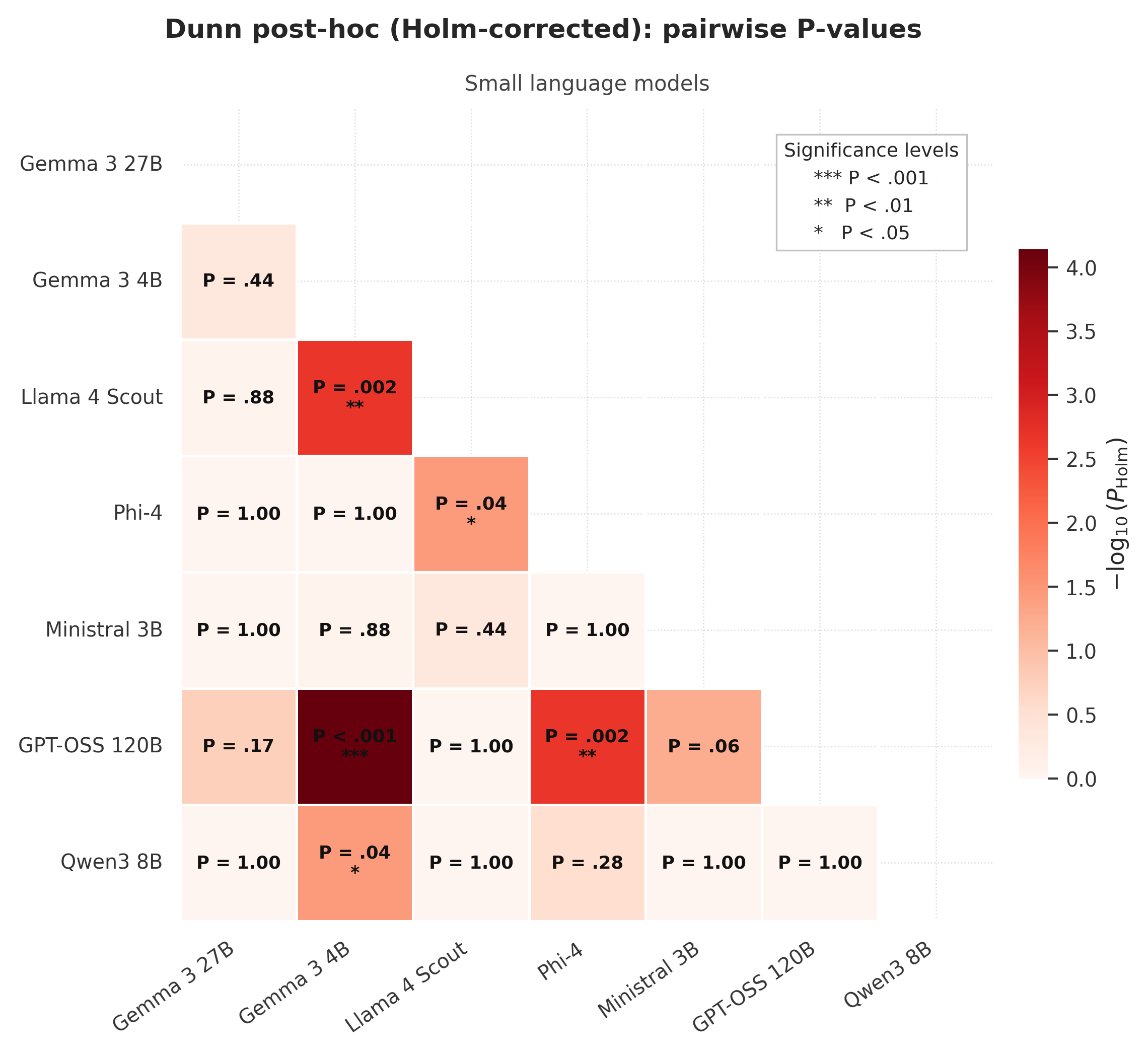


*Figure S11. Dunn post-hoc P-value heatmap (exploratory panel).*


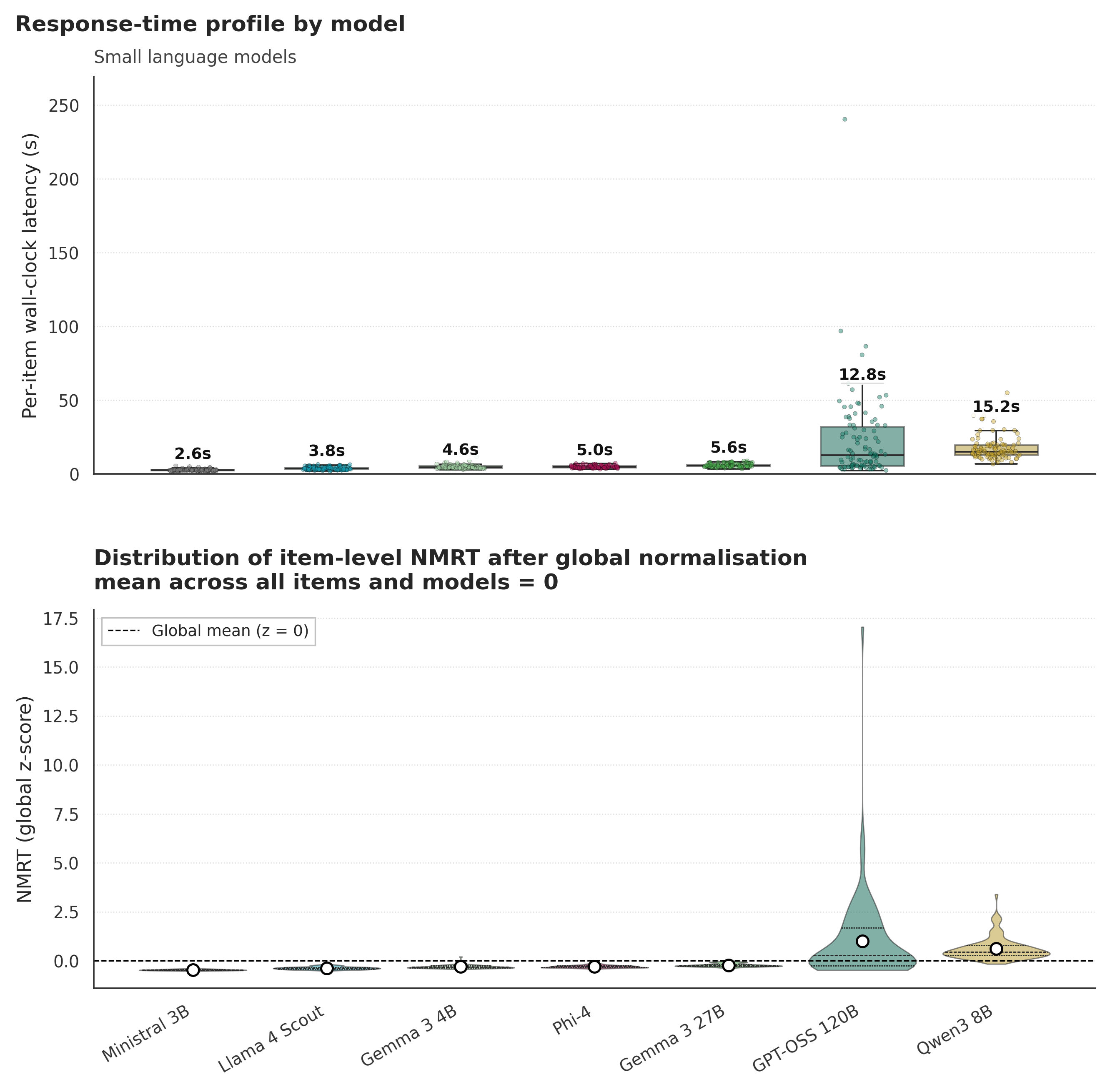


*Figure S12. NMRT violin distribution (exploratory panel).*


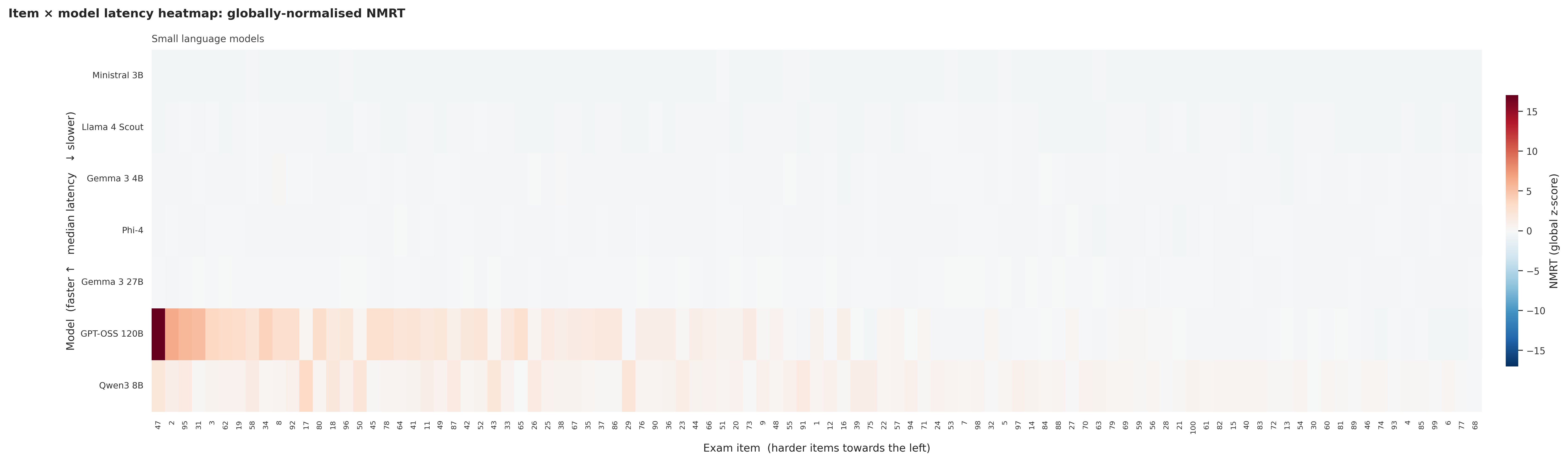


*Figure S13. NMRT per-item delta heatmap (exploratory panel).*


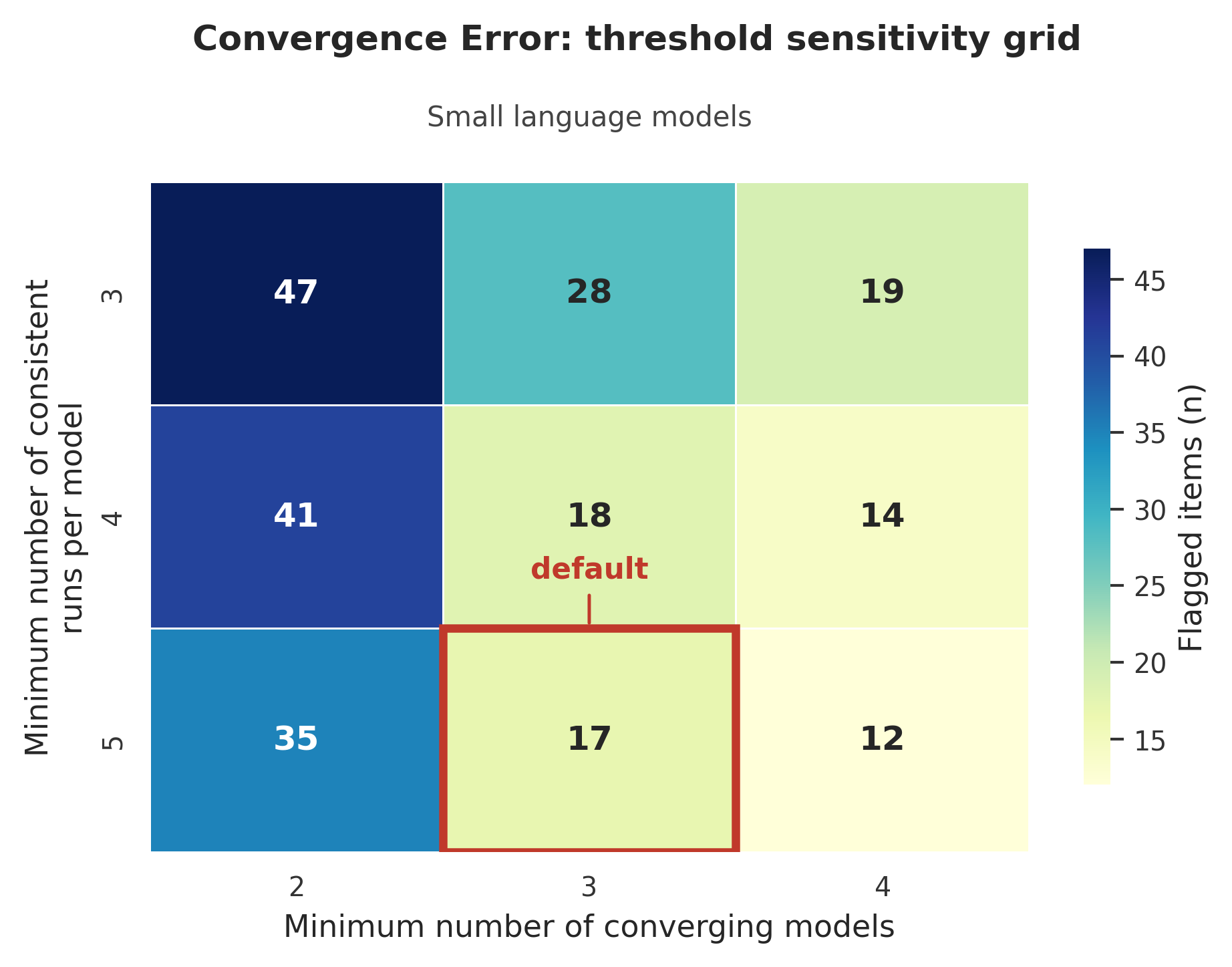


*Figure S14. CE sensitivity grid visualisation (exploratory panel).*
